# Machine learning for prediction of schizophrenia using genetic and demographic factors in the UK biobank

**DOI:** 10.1016/j.schres.2022.06.006

**Published:** 2022-08

**Authors:** Matthew Bracher-Smith, Elliott Rees, Georgina Menzies, James T.R. Walters, Michael C. O'Donovan, Michael J. Owen, George Kirov, Valentina Escott-Price

**Affiliations:** a MRC Centre for Neuropsychiatric Genetics and Genomics, Division of Psychological Medicine & Clinical Neurosciences, Cardiff University, UK; b School of Biosciences, Cardiff University, UK; c Dementia Research Institute, Cardiff University, UK

**Keywords:** Polygenic risk scores, Precision psychiatry, Schizophrenia, Machine learning

## Abstract

Machine learning (ML) holds promise for precision psychiatry, but its predictive performance is unclear. We assessed whether ML provided added value over logistic regression for prediction of schizophrenia, and compared models built using polygenic risk scores (PRS) or clinical/demographic factors.

LASSO and ridge-penalised logistic regression, support vector machines (SVM), random forests, boosting, neural networks and stacked models were trained to predict schizophrenia, using PRS for schizophrenia (PRS_*SZ*_), sex, parental depression, educational attainment, winter birth, handedness and number of siblings as predictors. Models were evaluated for discrimination using area under the receiver operator characteristic curve (AUROC) and relative importance of predictors using permutation feature importance (PFI). In a secondary analysis, fitted models were tested for association with schizophrenia-related traits which had not been used in model development.

Following learning curve analysis, 738 cases and 3690 randomly sampled controls were selected from the UK Biobank. ML models combining all predictors showed the highest discrimination (linear SVM, AUROC = 0.71), but did not significantly outperform logistic regression. AUROC was robust over 100 random resamples of controls. PFI identified PRS_*SZ*_ as the most important predictor. Highest variance in fitted models was explained by schizophrenia-related traits including fluid intelligence (most associated: linear SVM), digit symbol substitution (RBF SVM), BMI (XGBoost), smoking status (XGBoost) and deprivation (linear SVM).

In conclusion, ML approaches did not provide substantial added value for prediction of schizophrenia over logistic regression, as indexed by AUROC; however, risk scores derived with different ML approaches differ with respect to association with schizophrenia-related traits.

## 1 Introduction

Prediction modelling is more closely aligned with the aims of precision psychiatry than association testing (Bzdok et al., 2020) and raises the prospect of using supervised machine learning (ML), a collection of approaches which learn the relationship between predictors and response from data (Bzdok et al., 2018). ML can detect non-linear relationships, prioritises generalisation over drawing inference about a population from a sample, where generalisation refers to prediction in new individuals who were not included in model training, and may expedite the realisation of precision psychiatry by improving prediction from both genetic and non-genetic factors (Manchia et al., 2020).

There has been considerable interest in the use of polygenic risk scores (PRS) as a tool for prediction in psychiatry (Demontis et al., 2018; Levey et al., 2020; Mullins et al., 2021; Ripke et al., 2020; Vassos et al., 2017; Wray et al., 2018; Zheutlin et al., 2019). In schizophrenia, PRS currently explain around 8 % of the variance in liability in samples of European ancestry, and achieve moderate discrimination between cases and controls (0.72 area under the receiver operator characteristic curve; AUROC) (Ripke et al., 2020). Variance explained by PRS in samples of non-European ancestry is generally lower as the genome-wide association studies (GWAS) used to calculate PRSs are based predominantly on European samples (Dennison et al., 2020). PRS alone in schizophrenia do not have clinical utility (Vassos et al., 2017); useful prognostic models typically have AUROCs over 0.8 (Lewis and Vassos, 2020). Combining PRS with other predictors is a natural progression and has proved fruitful in both schizophrenia (Perkins et al., 2019) and outside psychiatry (Fung et al., 2019; Inouye et al., 2018), with most research to date using linear models rather than more flexible ML approaches.

Early ML applications in schizophrenia included prediction of clozapine response or weight changes as a result of medication using neural networks (Lan et al., 2008; Lin et al., 2008), where ML models using single nucleotide polymorphisms (SNPs) combined with demographic and lifestyle data improved prediction over logistic regression (LR). More recent work combining neuroimaging data with SNPs using ML has either not compared combined predictions with those from genetic or non-genetic data alone (Li et al., 2020; Pettersson-Yeo et al., 2013), has not found improved prediction from combined data types over only genetic or non-genetic predictors (Cao et al., 2013; Yang et al., 2010), or has found no added value from combining data types (Doan et al., 2017).

The potential benefit of machine learning over standard statistical approaches is unclear as performance estimates for ML may be overly optimistic (Boulesteix et al., 2013; Christodoulou et al., 2019; Hand, 2006; Whalen et al., 2021). Furthermore, we previously identified widespread high risk of bias (ROB) in genetic-only ML models in psychiatry, in addition to lack of comparison to LR or investigation of confounding by population structure (Bracher-Smith et al., 2020). Here, we aimed compare ML with LR, assess the relative importance of predictors and investigate model predictions for association with traits known to be associated with schizophrenia (referred to as schizophrenia-related traits hereafter). We mitigate previous issues in risk of bias by training ML approaches with low ROB strategies and assessing how well predictions are explained by population structure.

## 2 Material and methods

### 2.1 Participants

The UK Biobank contains around 500,000 participants which undertook cognitive assessments and physical measurements, provided blood samples, answered touch-screen questions and gave consent to participate (Sudlow et al., 2015). UK Biobank obtained informed consent from all participants; this study was conducted under approval from the NHS National Research Ethics Service (approval letter dated 13 May 2016, Ref 16/NW/0274) and under UK Biobank approvals for application number 13310. Unrelated individuals (kinship <0.04) who self-reported as white British or Irish (UK Biobank field 21,000) were selected for analysis to reduce confounding by population stratification. Genotypes were imputed by the UK Biobank (Bycroft et al., 2018); SNPs from the Haplotype Reference Consortium (HRC) were retained after quality control (Hardy-Weinberg equilibrium <10^−6^, minor allele frequency > 0.01, INFO >0.4, posterior probability >10^−4^).

342,512 participants were retained after exclusions. These were subsampled by schizophrenia status, which was derived using international classification of diseases (ICD)-10 codes for schizophrenia (codes F20.0-F20.9) or schizoaffective disorder (codes F25.0-F25.9) in hospital records (fields 41,202 and 41,204) or death records (fields 40,001 and 40,002), or if schizophrenia was self-reported, where inputs were verified by a trained nurse and only high-confidence classifications retained by UK Biobank (code 1289). PRS calculation requires independence of discovery and test sets. Due to the potential for identification of participants without permission, discovery and test datasets could not be formally de-duplicated. Individuals on clozapine (*n* = 52) were excluded from UK Biobank as they are potentially present in the discovery sample and can also be identified in UK Biobank without de-anonymising the data. We also note that as ours is a comparative study, the potential duplication of a small number of cases in the discovery GWAS and the test sample is not expected to result in better performance of ML over LR or vice versa. Individuals with other psychotic disorders (codes F21–23, F28, F29) or bipolar disorder (ICD-10 codes F30–31 and self-report code 1291), were excluded from the sample controls, resulting in 738 cases and 341,774 other individuals we consider here to be unaffected controls.

### 2.2 Predictors

As the objective is to assess ML models and the importance of genetic and non-genetic predictors, the standard pruning and thresholding (P + T) method was used for PRS calculation. The polygenic risk score for schizophrenia (PRS_*SZ*_) was created using a nominal (*p*_*T*_ = 0.05) *p*-value threshold, as it is the most predictive for schizophrenia (Pardiñas et al., 2018; Ripke et al., 2020, Ripke et al., 2014). SNPs were clumped (*r*^*2*^ = 0.2, distance 1 Mb), thresholded and combined into a PRS_*SZ*_ using effect sizes from the largest published peer-reviewed schizophrenia GWAS of predominantly European ancestry available at the time of the study (Pardiñas et al., 2018).

**Fig. 1:**
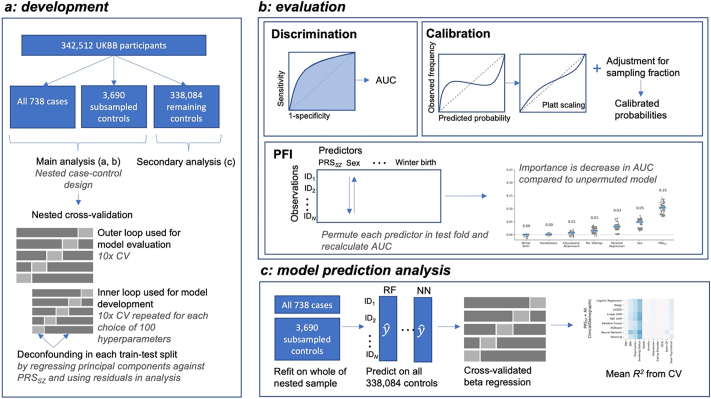
Overview of methodology. Analysis is comprised of model development (a), evaluation (b) and assessment of model predictions (c). A nested case-control design using a 1:5 ratio of cases-controls is used (a). Nested cross-validation is used as this separates model selection (on the inner loop) and evaluation (run for the outer loop), which gives a more accurate estimate of predictive performance than other approaches. Predictions from the outer round of cross-validation are used in assessing discrimination, calibration and permutation feature importance (PFI) (b). Models were then refit on the whole of the nested sample before predicting on remaining controls (c). A cross-validated beta regression was run with these predictions as the dependent variable and additional predictors, not used in any model development, as the independent variables, to assess how well the additional variables could explain the predictions. CV: cross-validation, UKBB: UK Biobank, PRS_*SZ*_: schizophrenia polygenic risk score, AUC: area under the receiver operator characteristic curve.

ML approaches have gained popularity in scenarios where the number of predictors is much greater than the sample size; however, their indiscriminate use in high-dimensional observational studies risks spurious associations or bias contributing to predictions if covariates are not correctly adjusted for. As such, we adjust genetic data for population structure and compare predictive models including 6 hand-selected clinical or demographic variables: sex (UK Biobank field 31), educational attainment (field 6138), season of birth (derived from field 52), severe parental depression (fields 20,107 and 20,110), number of siblings (fields 1883 and 1873) and handedness (field 1707). These were manually selected as they had evidence for association with schizophrenia (Davies et al., 2003; Dragovic and Hammond, 2005; MacCabe et al., 2008; McGrath et al., 2008; Radua et al., 2018; Wahlbeck et al., 2001), have mostly complete records, are easily collected and with the exception of severe depression in a parent that is relatively late in onset, are measurable before onset of schizophrenia in most individuals. Schizophrenia-related traits which are likely to occur or be measured after onset, such as performance on cognitive tests, were included in a secondary analysis assessing the relevance of resulting ML models (which are built to predict schizophrenia) to known schizophrenia-related traits (Fig. 1c). These traits were not used for building the ML models predicting schizophrenia (ML_*SZ*_). Several additional factors widely considered to increase risk for schizophrenia (e.g. obstetric complications and drug use) could not be included as they were unavailable in UK Biobank, or the data were substantially missing. Coding for sex, educational attainment (split at General Certificate of Secondary Education (GCSE), the standard qualification in a school subject typically taken at 15 or 16 years old), handedness (left and ambiguous grouped) and winter birth (winter as December to February, inclusive) were binary. Number of full brothers (field 1873) and number of full sisters (field 1883) were combined to create number of siblings, truncated at 10 and log transformed. Severe parental depression was derived from illness in the mother (20110) or father (20107), as selected by participants from a list of illnesses under supervision by a trained nurse, and coded as 0, 1 or 2 for the number of parents affected.

### 2.3 Model development and evaluation

The main analyses assessed discrimination and calibration using a nested case-control design of 1:5 cases to randomly sampled controls, following recommendations (Biesheuvel et al., 2008) and a learning curve analysis (Fig. S8), as this greatly reduces computational burden. Participants with missingness were excluded before sampling as imputation within all rounds of cross-validation for all classifiers was computationally infeasible. Training was undertaken by 10-fold nested cross-validation (Vabalas et al., 2019; Varma and Simon, 2006), a resampling approach where training data are divided into 10 train-test set pairs, with models refit in each training split, or fold, and evaluated in its corresponding test split (Fig. 1). Hyper-parameters were tuned using 100 iterations of random search (Bergstra and Bengio, 2012). To adjust for the linear effects of confounders during model development, principal components and genotyping array provided by UK Biobank were regressed against PRS_*sz*_ within each fold of cross-validation, with the residuals forming the new predictors (see Appendix A). This procedure is referred to as deconfounding.

Discrimination between cases and controls was assessed using the median area under the receiver operator characteristic curve (AUROC) and area under the precision-recall curve (AUPRC) from cross-validation. Classifiers were compared using the Wilcoxon signed-rank test (Demšar, 2006; Dietterich, 1998), with multiple testing accounted for using Benjamini-Hochberg false discovery rate (FDR) at 0.05. ML_*SZ*_ models were re-calibrated using Platt scaling (Platt and Platt, 1999) to allow for fair comparison of predicted probabilities, as tree-based models such as random forests and gradient boosting force probabilities to be less extreme (Niculescu-Mizil and Caruana, 2005), and support vector machines (SVMs) output distance from the hyperplane which can lie outside the unit interval. Calibration, which indicates how well predicted probabilities align with the observed frequency of schizophrenia, was assessed graphically (Austin and Steyerberg, 2014).

### 2.4 Predictor importance

Permutation feature importance (PFI) scores were used to assign a model-agnostic measure of relative importance to predictors that enabled consistent interpretation across models (Breiman, 2001; Molnar, 2019). While AUROC describes the ability of the model to discriminate between cases and controls, PFI indicates which predictors are most important to achieving that discrimination. Each predictor was permuted and used to re-generate predictions in each fold of cross-validation. The average drop in discrimination compared to the non-permuted model defines the importance score. Permutations were also implemented in a group-wise manor, as applied in deep learning (Kokhlikyan et al., 2020), where types of predictors were shuffled together, giving an estimate of the relative importance of genetic and clinical/demographic factors taken as a whole.

### 2.5 Association with schizophrenia-related traits and deconfounding

In a secondary analysis, predictions from the best performing ML models (also known as “fitted values” in regression analyses) were further validated by investigating which additional variables, which were not used for the model construction, they were associated with. Since all schizophrenia cases available in the UK Biobank were used to build the ML_*SZ*_ models (to achieve maximal power), the remaining controls and schizophrenia-related traits were used. In this sample of additional controls, we first calculated individuals' fitted values obtained by the derived ML_*SZ*_ models, which were built to predict schizophrenia using a combination of PRS_*SZ*_, sex, parental depression, educational attainment, winter birth, handedness and number of siblings. We then investigated which variables are associated with these fitted values in the remaining 338,084 controls. These analyses were run using a 5-fold cross-validated beta regression for the assessment, as described elsewhere (Kohoutová et al., 2020) (see Appendix A).

Principal components and genotyping array platform were used as the additional variables to evaluate the deconfounding procedures used in model development, while cognitive tests, neurological diseases, psychiatric disorders, and additional demographic variables which occur after onset (Table S1) were also used to assess how well predictions captured schizophrenia-related traits.

### 2.6 Algorithms

Ridge (Hoerl and Kennard, 1970) and least absolute shrinkage and selection operator (LASSO) (Tibshirani, 1996) regression were assessed by applying the LASSO (*L*_*1*_) and ridge (*L*_*2*_) penalties to logistic regression to shrink coefficient estimates. Support vector machines (SVMs) apply a kernel-based approach to learn a maximally-separating hyperplane (Cortes and Vapnik, 1995). Linear and radial basis function (RBF) kernels were applied, where the latter allows for non-linear decision boundaries to be learned in higher-dimensional space (Noble, 2006).

Random forests and gradient boosting combine greedy decision trees that perform recursive binary splits to partition the data (Breiman, 1984). While random forests average over deeper trees to reduce variance, boosting sequentially adds weak learners to reduce bias (Friedman, 2001). Gradient boosting was implemented using the highly-optimised eXtreme Gradient Boosting (XGBoost) package (Chen and Guestrin, 2016).

Neural networks were utilised through a fully-connected feed-forward multilayer perceptron, trained to apply a network of weights which are learned iteratively through backpropagation (LeCun et al., 2015). Models were assessed individually and as an ensemble using stacking, which “stacked” predictions from base estimators to use as predictors in a logistic regression meta-estimator (Wolpert, 1992). All models were further compared to unpenalised logistic regression on the original predictors. Hyperparameter tuning is described further in Appendix A (Fig. S1).

### 2.7 Implementation

Cross-validation used the same random seed for all train-test splits, including neural networks implemented in PyTorch, with all transformations conducted in scikit-learn pipelines to avoid information ‘leakage’ and ensure reproducibility. Classes used in nested cross-validation were adapted to allow for regressing-off principal components within cross-validation using a deconfounding scikit-learn transformer. Analyses were run using the Python scientific computing stack (Harris et al., 2020; Hunter, 2007; Mckinney, 2010; Pedregosa et al., 2011; Virtanen et al., 2020) and the Cardiff Hawk supercomputer; neural networks were run on Nvidia V100 and P100 graphical processing units (GPUs).

## 3 Results

### 3.1 Sample

342,512 participants were included following exclusions and filtering for missingness. Controls were randomly subsampled to give a 1:5 nested case-control study design of 738 cases and 3690 controls. Examination of observations before and after missingness filters and subsampling indicate the analysed subsample is representative of the larger UK Biobank cohort (Figs. S2–S4; Table S2).

### 3.2 Model performance

**Fig. 2:**
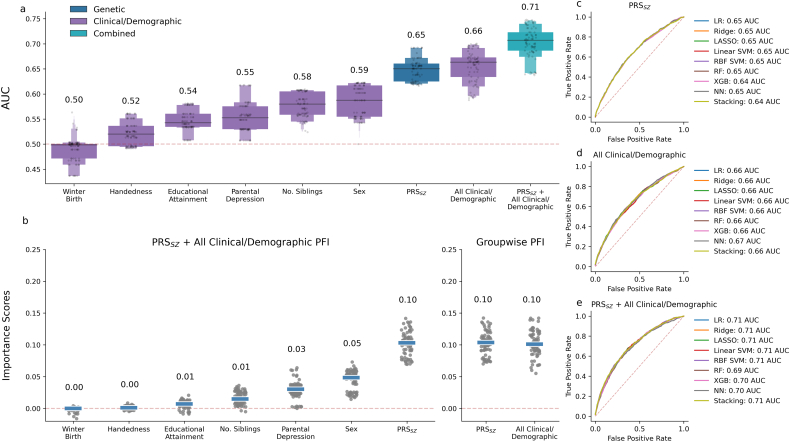
Discrimination and importance scores across. Boxen plots of pooled test fold AUROCs from cross-validation for all classifiers show best prediction from combined predictors compared to each predictor individually (a). Median per-predictor permutation feature importance (PFI) scores (b, left) across folds for all classifiers gives sex and schizophrenia polygenic risk score (PRS_*SZ*_) as the strongest predictors, while per-group importance (b, right) shows PRS_*SZ*_ is similar in importance to all clinical and demographic predictors taken together. Importance scores do not indicate direction of effect, for which estimates are given in Appendix B (Table S3). Average receiver operator characteristic (ROC) curves show similar average discrimination across classifiers in models using only PRS_*SZ*_ (c) or all clinical/demographic predictors (d) individually or in combination (e). LR: logistic regression, LASSO: least absolute shrinkage and selection operator, RF: random forest, SVM: support vector machine, RBF: radial basis function, XGB: XGBoost, NN: neural network. Discrimination and importance scores across. Boxen plots of pooled test fold AUROCs from cross-validation for all classifiers show best prediction from combined predictors compared to each predictor individually (a). Median per-predictor permutation feature importance (PFI) scores (b, left) across folds for all classifiers gives sex and schizophrenia polygenic risk score (PRS_*SZ*_) as the strongest predictors, while per-group importance (b, right) shows PRS_*SZ*_ is similar in importance to all clinical and demographic predictors taken together. Importance scores do not indicate direction of effect, for which estimates are given in Appendix B (Table S3). Average receiver operator characteristic (ROC) curves show similar average discrimination across classifiers in models using only PRS_*SZ*_ (c) or all clinical/demographic predictors (d) individually or in combination (e). LR: logistic regression, LASSO: least absolute shrinkage and selection operator, RF: random forest, SVM: support vector machine, RBF: radial basis function, XGB: XGBoost, NN: neural network.

Across all modelling approaches, all variables had a median AUROC above 0.5 apart from winter birth (Fig. 2a). Weak discrimination was observed for individual clinical/demographic predictors (0.5–0.59 median AUROC across all modelling approaches). Moderate discrimination was achieved by models using all clinical/demographic predictors together and those using PRS_*SZ*_ alone (0.65–0.67 AUROC). Approaches which combined PRS_*SZ*_ and all clinical/demographic predictors attained good discrimination (0.71 AUROC) (D’Agostino et al., 2013). Tests comparing AUROC provide strong evidence that models developed using a combination of PRS_*SZ*_ and all clinical/demographic predictors show better discrimination than either alone (Table 1). Median importance scores were similar across models (Fig. S5); models which combined genetic and demographic predictors assigned no importance to handedness and low importance to educational attainment (Fig. 2b). Sex and PRS_*SZ*_ were ranked highest, with PRS_*SZ*_ and all demographic predictors roughly equal in their contribution to group-wise importance scores. The validity of this approach was demonstrated through inclusion of noise predictors, which were attributed importance scores of zero (Fig. S6). As importance scores do not show direction of effect, a multivariable logistic regression was fit in the nested sample, adjusted for (genetic) principal components and genotyping array (Table S3). This regression analysis showed no association of schizophrenia with winter birth, whereas non-right handedness, lower educational attainment, higher number of siblings, being male, presence of parental depression and higher PRS_*SZ*_ were shown to be associated with higher risk of schizophrenia, consistent with previous studies (Table S3).

**Table 1:** Statistical comparison of models. Consistently low test-statistics and *p*-values indicate strong evidence that models developed using a combination of schizophrenia polygenic risk score (PRS_*SZ*_) and all clinical/demographic variables are better able to discriminate between case and controls than models built using only PRS_*SZ*_ or clinical/demographic variables alone. Difference in area under the receiver operator characteristic curve (AUROC) indicates how much higher the AUROC is for each modelling approach when using all predictors combined compared to either genetic or non-genetic alone**.** The test statistic for the Wilcoxon signed rank test, *W*, is given for all comparisons of the AUROC from each outer test fold of nested cross-validation, split by classifier and dataset. Comparisons have a *W* of 0 as all corresponding test folds for the combined models have a higher AUROC. *P*-values are FDR-corrected at 0.05; starred adjusted *p*-values are significant at the 5 % level.

Modelling approach	Comparison	*W*	*p*	*Difference in % AUROC*
Logistic regression	Combined vs. PRS_*SZ*_	0	0.005*	5.67
Logistic regression	Combined vs. clinical/demographic	0	0.005*	4.68
Ridge	Combined vs. PRS_*SZ*_	0	0.005*	5.67
Ridge	Combined vs. clinical/demographic	0	0.005*	4.68
LASSO	Combined vs. PRS_*SZ*_	0	0.005*	5.81
LASSO	Combined vs. clinical/demographic	0	0.005*	4.86
Linear SVM	Combined vs. PRS_*SZ*_	0	0.005*	5.97
Linear SVM	Combined vs. clinical/demographic	0	0.005*	5.35
RBF SVM	Combined vs. PRS_*SZ*_	0	0.005*	5.56
RBF SVM	Combined vs. clinical/demographic	0	0.005*	4.49
Random forest	Combined vs. PRS_*SZ*_	0	0.005*	4.03
Random forest	Combined vs. clinical/demographic	0	0.005*	3.48
XGBoost	Combined vs. PRS_*SZ*_	0	0.005*	5.79
XGBoost	Combined vs. clinical/demographic	0	0.005*	4.50
Neural network	Combined vs. PRS_*SZ*_	0	0.005*	5.44
Neural network	Combined vs. clinical/demographic	0	0.005*	3.24

The highest AUROCs were achieved by machine learning models when developed using only PRS_*SZ*_ (best ML model: random forest, 0.65 AUROC), clinical/demographic factors (neural network, 0.67 AUROC) or all predictors combined (linear SVM, 0.71 AUROC). Best-performing ML approaches had higher AUROC than logistic regression, but hypothesis testing found the differences were not statistically significant (*p* = 0.17, 0.58 and 0.65, for PRS_*SZ*_, clinical/demographic and all predictors, respectively). Discrimination was similar between machine learning approaches, as shown by overlying average receiver operator characteristic curves (Fig. 2c, d and e). Similarity of AUROC between classifiers was robust over 100 iterations of repeated resampling of the controls, showing highly overlapping confidence intervals (Fig. S7). AUROC was also stable when varying the sampling fraction of controls in a learning curve analysis (Fig. S8). Discrimination assessed by area under the precision-recall curve (AUPRC), which may be more useful than AUROC under severe class imbalance (Saito and Rehmsmeier, 2015), was also highly similar between classifiers (Table S4). Calibration, the alignment of predicted probabilities and observed frequencies of schizophrenia, was good for all models after Platt scaling and adjusting for the sampling fraction (Figs. S9–S14).

### 3.3 Association with schizophrenia-related traits and deconfounding

**Fig. 3:**
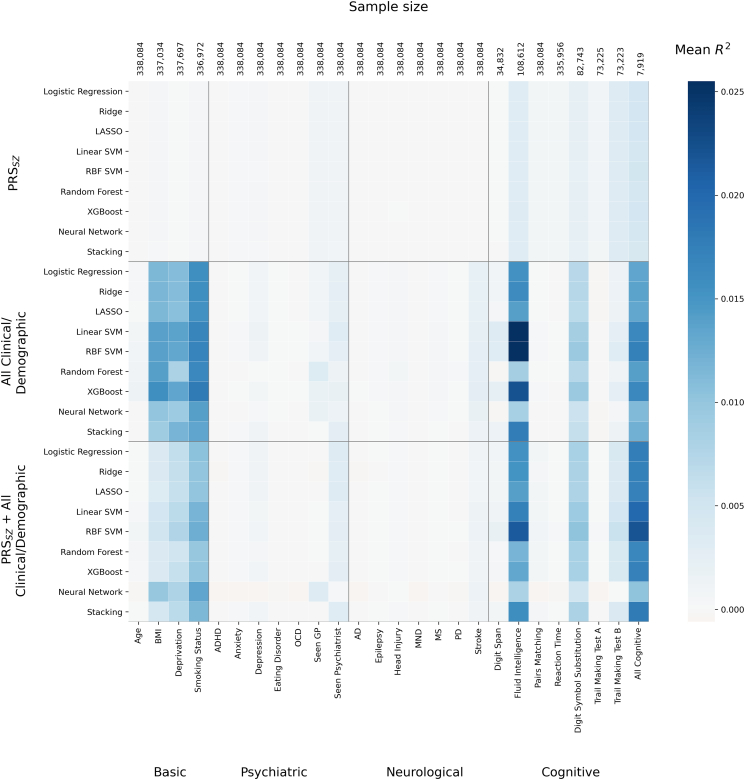
Generalisable associations of model predictions. 5-fold cross-validation of a beta regression in all remaining controls. Features on the x-axis were independent variables, and calibrated model predictions of schizophrenia from each method on the y-axis were the dependent variable*.* Each tile in the heatmap therefore indicates how well the variable on the x-axis explains the predictions of schizophrenia which were generated using the corresponding predictors and modelling approach annotated on the y-axis. Cross-validation is used to assess modelling under a prediction modelling paradigm which emphasises generalisation; the darker blue tiles show mean test-fold *R*^*2*^, and so indicate which variables on the x-axis explain predictions in new observations, not simply the training data. Variation in tiles in a vertical line, such as for fluid intelligence, highlight how the fitted ML models vary in how well they are explained by additional variables, despite being trained on the same predictors. Seen GP or psychiatrist refer to ever having seen either for “nerves, anxiety, tension or depression”. Other variables are described in the Appendix A. AD: Alzheimer's disease, ADHD: attention deficit hyperactivity disorder, BMI: body mass index, MND: motor neurone disease, MS: multiple sclerosis, OCD: obsessive compulsive disorder, PD: Parkinson’s disease. (For interpretation of the references to colour in this figure legend, the reader is referred to the web version of this article.) Generalisable associations of model predictions. 5-fold cross-validation of a beta regression in all remaining controls. Features on the x-axis were independent variables, and calibrated model predictions of schizophrenia from each method on the y-axis were the dependent variable*.* Each tile in the heatmap therefore indicates how well the variable on the x-axis explains the predictions of schizophrenia which were generated using the corresponding predictors and modelling approach annotated on the y-axis. Cross-validation is used to assess modelling under a prediction modelling paradigm which emphasises generalisation; the darker blue tiles show mean test-fold *R*^*2*^, and so indicate which variables on the x-axis explain predictions in new observations, not simply the training data. Variation in tiles in a vertical line, such as for fluid intelligence, highlight how the fitted ML models vary in how well they are explained by additional variables, despite being trained on the same predictors. Seen GP or psychiatrist refer to ever having seen either for “nerves, anxiety, tension or depression”. Other variables are described in the Appendix A. AD: Alzheimer's disease, ADHD: attention deficit hyperactivity disorder, BMI: body mass index, MND: motor neurone disease, MS: multiple sclerosis, OCD: obsessive compulsive disorder, PD: Parkinson’s disease. (For interpretation of the references to colour in this figure legend, the reader is referred to the web version of this article.)

In a secondary analysis (Fig. 3), fitted machine learning models predicting schizophrenia (ML_*SZ*_) were assessed for association with schizophrenia-related traits using a cross-validated beta regression (described in Section 2.5 and Appendix A). In this analysis, predicted risk assigned by ML_*SZ*_ differed in their association with known SZ-related traits. Fig. 3 illustrates that fluid intelligence had the greatest variability in how well it explained predictions from fitted models. Predictions from SVMs, for example, were better explained by fluid intelligence than predictions from random forests and neural networks, despite all having similar discrimination between cases and controls (AUROC). Relatively high variance in predicted risk from fitted models was also explained by a higher chance of smoking, greater deprivation, higher body mass index (BMI) and worse cognitive performance (Table S5). Analysis of other psychiatric disorders and neurological diseases found little or no association with risk scores from the fitted ML and logistic regression (LR) models (Fig. 3).

The same methodology was used in the remaining controls to assess how well deconfounding procedures used in model development had removed the linear effect of confounders from the predictors (Fig. S15). Elevated mean *R*^*2*^ was present for clinical/demographic-only (maximum *R*^*2*^ = 0.012, XGBoost) and combined models (*R*^*2*^ = 0.0078, neural networks) which include non-genetic predictors that were not adjusted for principal components or genotyping array.

## 4 Discussion

Discrimination between schizophrenia cases and controls using each predictor individually demonstrated almost all variables had better than chance prediction, yet permutation-based importance measures showed low importance in joint models for handedness (0.52 median AUROC, 0 median importance) and educational attainment (0.54 AUROC, 0.01 importance). Results also suggest that some demographic predictors may be redundant. For instance, prediction from number of siblings alone achieves a median of 0.58 AUROC, yet the median decrease in AUROC from permuting it in a multivariable model is low at 0.02–0.03. By contrast, parental depression has a similar decrease in AUROC from its permutation (0.03–0.04 AUROC) to what is predicted using it alone (0.55 AUROC; i.e. 0.05 above chance), indicating it is more independent from the other factors in the model than is number of siblings.

Similar AUROC was reported for models using either PRS_*SZ*_ or all clinical/demographic variables, yet combined models have around 5 % higher AUROC, suggesting the information from these two sources is partially independent. This is consistent with previous findings that PRS_*SZ*_ and environmental exposures interact additively (Guloksuz et al., 2019); however, recent work in schizophrenia has indicated that PRS does not provide additional information over clinical predictors obtained in a psychiatric interview, including symptoms, family history of schizophrenia, sex, and other factors, for prediction of poor outcomes (Landi et al., 2021). Our results suggest that PRS_*SZ*_ may be a useful addition to prediction of schizophrenia in large cohorts where basic clinical and demographic factors are widely available, which are more easily collected and occur mainly before disorder onset. The predictors included in our ML_*SZ*_ models are not exhaustive and their relative contribution to models may change with inclusion of more factors such as those related to prenatal and perinatal events, early adversity and drug use. Comparison of PRS methods has also indicated that use of LDPRED2, SBayesR and MegaPRS, which perform similarly to each other, may improve prediction of schizophrenia by around 2–3 % AUROC, compared to the most frequently used pruning and thresholding (P + T) approach used here (Ni et al., 2021; Zhou and Zhao, 2021). AUROC in the current analysis may therefore increase slightly if using PRS approaches which aim to formally model genetic architecture; however, as the focus here was comparison between approaches, the relative discriminative ability of ML approaches would likely remain unchanged. Similarly, a wide array of ML approaches exist, and others such as CatBoost and LightGBM may also slightly augment performance.

Examination of model predictions assesses how well additional variables of interest (e.g. confounders or consequent variables) can explain model predictions, and has been recommended as standard practice in machine learning (Kohoutová et al., 2020). This was implemented here through cross-validation in population-based controls which were not used for training and assessing discrimination (Fig. 3), and highlighted known associations with schizophrenia including fluid intelligence and processing speed (as measured by digit symbol substitution), in addition to BMI, social deprivation and smoking status. Differences between modelling approaches shown in Fig. 3 may be important for clinical applications, as heterogeneity in how models weight input data means predictions by different modelling approaches show variation in their association with outcome-related factors. This highlights the importance of moving beyond simple scalar summaries of model performance and assessing prediction of outcome-related variables. Caution should be taken in interpretation of these, however, as a focus on prediction precludes use of covariates, meaning that a higher *R*^*2*^ may be partially explained by other variables not included in the beta regression model. The technique highlights which variables are associated with model predictions of schizophrenia, not schizophrenia itself; the low *R*^*2*^ for psychiatric phenotypes simply indicate they explain little variance in model predictions, and therefore does not contradict known genetic and phenotypic correlations between phenotypes themselves. Our results also suggest that current deconfounding procedures which regress-off principal components from predictors do not remove all effects of population structure from the final predictions, particularly when including unadjusted non-genetic factors in models; alternative deconfounding procedures may be required for machine learning (Chyzhyk et al., 2018; Dinga et al., 2020; Zhao et al., 2020). This analysis also serves as a minimal test of generalisation by using an independent subsample within the UK Biobank. Though ideally results would be replicated in a fully external dataset, confidence in the generalisability of models is added by stability of results across classifiers, metrics and resampling of controls, consistency of results with expected direction of effects for associations with schizophrenia and schizophrenia-related traits, and the use of low risk of bias strategies such as nested cross-validation.

The size of the full UK Biobank dataset raises issues for complex machine learning models which can be computationally intensive. We show that the nested case-control study is an efficient design for applying ML methods to large cohorts under reduced computational burden, with discrimination stable across sampling fractions, and a large sample of remaining controls left available for evaluating predictions, for which computation is cheap. Further, we show that concerns of inflated performance estimates (Bracher-Smith et al., 2020; Christodoulou et al., 2019) can be mitigated through low ROB model development strategies.

Volunteer bias in the UK Biobank means the dataset in general is less socioeconomically deprived, healthier and more likely to be female and white British than the UK population as a whole (Fry et al., 2017), and individuals with the most severe forms of schizophrenia may be underrepresented or even absent. Ascertainment biases may cause effect sizes to differ if estimated in a more representative sample, with potential consequences for discrimination and calibration that would mean models require further shrinkage of coefficients, recalibration or retraining before use in the general population or a clinical target sample.

## 5 Conclusions

In conclusion, our results suggest that while the diversity of modelling procedures in ML may yet prove to be useful in precision psychiatry, they do not currently provide added benefit through improved discrimination between schizophrenia cases and controls. We show however that schizophrenia risk scores derived with different ML_*SZ*_ approaches show a non-homogeneous pattern of association with traits that are known to be related to schizophrenia.

## Role of the funding source

Funding sources played no role in analysis, reporting or submission of results.

## Declaration of competing interest

None.

## Appendices Supplementary data

Appendix A Supplementary methods. Appendix A

Appendix B Supplementary results. Appendix B
