# Supplementary material for: Machine learning for prediction of schizophrenia using genetic and demographic factors in the UK biobank
Source: Schizophr Res. 2022 Aug;246:156–64. doi: 10.1016/j.schres.2022.06.006 (PMC9399753; doi:10.1016/j.schres.2022.06.006)
Supplement: Appendix A — Supplementary methods. [file mmc1.docx]

## Appendix A: supplementary methods

#### Hyperparameter tuning

SVMs are parameterised by hyper-parameters *C*, which controls violations to the margin either side of the hyperplane, and gamma (RBF SVMs only) which affects the influence of more distant observations on the decision boundary.

Random forests were grown using the Gini index and 1000 trees were tuned for tree depth, predictors at each split, and minimum observations in child nodes. XGBoost was run using 1000 boosting rounds, with tree depth*, L_2_* penalty, predictor subsampling, and learning rate tuned during training

For neural networks a multilayer perceptron was trained with rectified linear unit (ReLU) activation functions, He initialisation^1^, *L_2_* weight decay, mini-batch gradient descent with momentum and batch norm^2^. Batch size, momentum and epochs were fixed at 32, 0.9 and 15 respectively; learning rate, *L_2_* penalty, number of hidden layers and number of units per hidden layer were chosen by cross-validation.

Hyperparameters which were chosen through cross-validation (CV) were assessed in the inner fold of nested CV, which was repeated 100 times, each time drawing a different selection of hyperparameters from the chosen distributions. Distributions are shown in Figure S1.


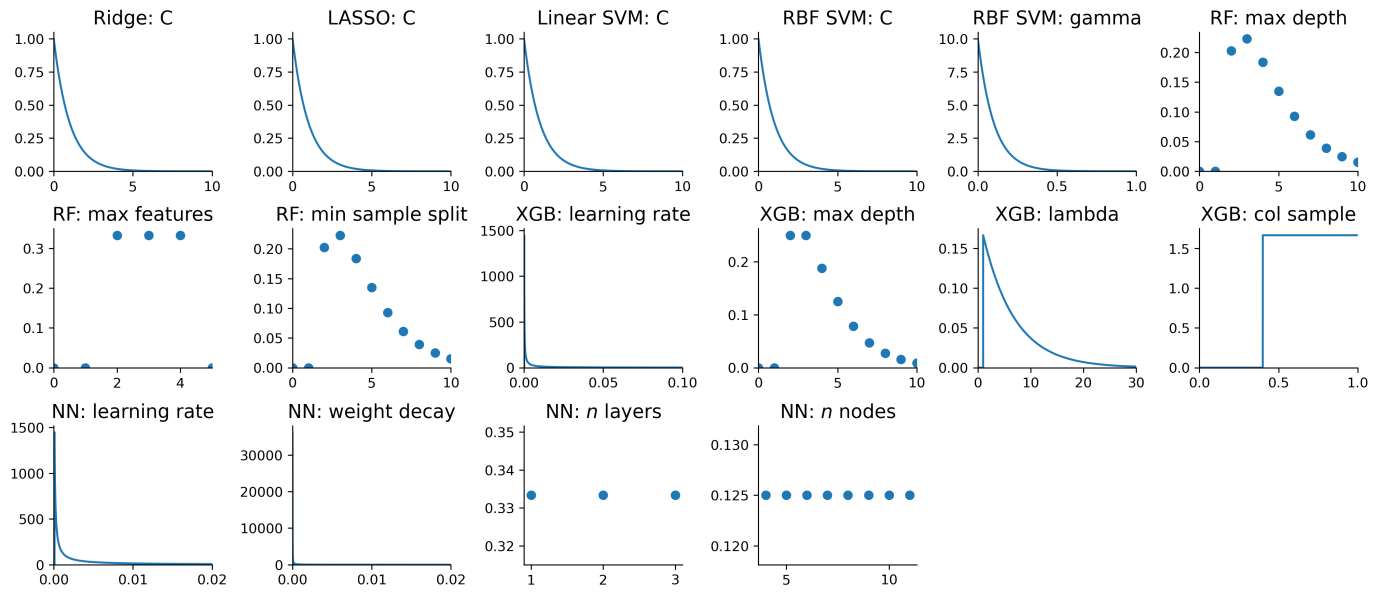


**Figure S1: Distributions for random search of hyperparameters**. Examples are given for models when trained on a combined dataset or PRS and demographic predictors. Probability density functions (PDFs) and probability mass functions (PMFs) are shown for machine learning methods.

#### Calibration

For all measures of discrimination or importance, values were generated separately on each test fold from nested cross-validation, with the median reported. For graphical calibration, to allow for simpler visualisations, predictions from test folds were combined.

Platt scaling was performed within cross-validation by taking the test-fold predictions from the inner round of CV which was run using the set of hyperparameters which achieved the best performance. The test-fold predictions were stacked together to form the training data for a logistic regression, with the original test-fold prediction from outer CV forming the test-set input data to the logistic regression. This follows the steps described by Platt et al. for rescaling predictions for better calibration without the high risk of overfitting which is present when just using training-fold predictions from outer CV as the input to logistic regression^3^.

To assess calibration in the whole dataset, which gives an indication of how well predicted probabilities correspond to actual chances of schizophrenia, predictions were obtained by re-training models on the nested subsample before predicting in remaining controls which were not previously included when subsampling, and applying Platt scaling to this process as previously described. These were combined with predictions from nested CV, so all observations were assigned a risk score by each classifier. The resulting probabilities were adjusted for the sampling fraction used in the nested design using Elkan’s transformation^4^ which accounts for differing base rates (prevalence) between samples.

#### Beta regression

Model predictions were analysed for how well they could be predicted by additional schizophrenia-related variables. This assessed which aspects of schizophrenia-related traits were captured by different modelling approaches. Under an explanatory modelling paradigm, this may be conducted using association tests, with model predictions as the dependent variable and additional variables as interested as the independent variables, with effect sizes reported. Here, we focus on a prediction modelling context, so use cross-validation and report the mean test-fold *R^2^* for prediction. As predicted probabilities are constrained to the unit interval, we use a beta regression to appropriately model the dependent variable. Factors used as independent variables are given in Table S1.

| **Variable** | **Fields/Codes** | **Description** | **Handling** |
| --- | --- | --- | --- |
| Age | Field 22148 | Age at recruitment | None |
| BMI | Field 21001 | Body mass index | Log transform |
| Deprivation | Field 189 | Townsend deprivation index at recruitment | Log transform |
| Smoking status | Field 20116 | Current/past smoking status | Previously smoked and currently smoke combined to give variable of 1 if smoked and 0 if never smoked |
| ADHD | F90 | Attention deficit hyperactivity disorder | 1 if present, 0 if absent |
| Anxiety | F43, 1287 |  | 1 if present, 0 if abse1 if present, 0 if absent nt |
| Depression | F32, F33, 1286 |  | 1 if present, 0 if absent |
| Eating disorder | F50, 1470 |  | 1 if present, 0 if absent |
| OCD | F42, 1615 | Obsessive compulsive disorder | 1 if present, 0 if absent |
| Seen GP | Field 2090 | Seen a general practitioner for nerves, anxiety, tension or depression | 1 if present, 0 if absent |
| Seen psychiatrist | Field 2100 | Seen a psychiatrist for nerves, anxiety, tension or depression | 1 if present, 0 if absent |
| AD | G30 | Alzheimer’s disease | 1 if present, 0 if absent |
| Epilepsy | G40, G41, 1264 |  | 1 if present, 0 if absent |
| Head injury | 1266 |  | 1 if present, 0 if absent |
| MND | G122, 1259 | Motor neurone disease | 1 if present, 0 if absent |
| MS | G35, G36, G37, 1261 | Multiple sclerosis | 1 if present, 0 if absent |
| PD | G20, G21, G22, 1262 | Parkinson’s Disease | 1 if present, 0 if absent |
| Stroke | I60, I61, I62, I63, I64, I66, I67, I68, I69, 1081, 1082, 1083, 1086, 1491, 1583 |  | 1 if present, 0 if absent |
| Digit span | Field 4282 | Maximum digits correctly recalled in numeric memory assessment | Abandoned tests coded as missing |
| Fluid intelligence | Field 20016 | Sum of correct fluid intelligence questions | Values in interval (0, 13), assigned 0 if questions not completed |
| Pairs matching | Field 399 | Number of correct matches | Log (1+p) transform |
| Reaction time | Field 20023 | Average time to identify matches | None |
| Digit symbol substitution | Field 23324 | Number of symbol digit matches made correctly | None |
| Trail making test A | Field 6348 | Completion time for numerically-ordered trail test | Log transform |
| Trail making test B | Field 6350 | Completion time for alphabetically-ordered trail test | Log transform |
| All cognitive | See above | All cognitive tests above in a multivariable model | See above |
| Principal components | Field 22009 | Genetic principal components 1-15 provided by UK Biobank | None |
| Genotyping array | Field 22000 | Measurement batch as 1 or 0 for BiLEVE or Axiom, provided by UK Biobank | None |

**Table S1: Variables used to predict model outputs in remaining controls**. Codes given for diseases and disorders were search for matches in fields 41202.0.0 - 41202.0.65 for primary records, and 41204.0.0 - 41204.0.183 for secondary records. Death records were searched in 40001.0.0 for primary cause of death, and 40002.0.0 - 40002.0.13 for secondary. Self-report codes were checked in fields 20002.0.0 - 20002.2.33. Codes beginning with a letter are ICD-10; numeric as self-report.

#### Deconfounding

Deconfounding describes procedures which attempt to remove the effects of confounding variables. This is typically done by regressing-off the linear effects of a confounder from predictors before analysis^5^. While reasonable in an explanatory modelling context where association is tested in the whole dataset, here cross-validation is used to assess models. This means running deconfounding on the entire dataset before CV does not fit with prediction as it assumes all data, including test data, are available in advance, and so is not feasible where prediction on new observations is expected. In addition, it breaks a core assumption of cross-validation that observations are independent, and the assumption of most machine learning methods that observations are independent and identically distributed (IID).

We therefore implemented deconfounding within cross-validation, as recommended elsewhere^6^. This used principal components and genotyping array provided by UK Biobank (Table S1). Estimating coefficients for the effect of confounders on predictors was run in the training fold in each train-test fold pair, with these coefficients use to derive residuals for both the train and test set. This avoids refitting coefficients in the test data separately, which would give a much smaller sample in which to estimate the effects of confounders and would again assume all predictions could only be done on new batches of data, rather than on new individuals which may appear one-at-a-time.

#### References

1. He K, Zhang X, Ren S, Sun J. Delving Deep into Rectifiers: Surpassing Human-Level Performance on ImageNet Classification. February 2015. http://arxiv.org/abs/1502.01852. Accessed April 22, 2019.

2. Ioffe S, Szegedy C. Batch normalization: Accelerating deep network training by reducing internal covariate shift. In: *32nd International Conference on Machine Learning, ICML 2015*. Vol 1. International Machine Learning Society (IMLS); 2015:448-456. https://arxiv.org/abs/1502.03167v3. Accessed July 1, 2020.

3. Platt JC, Platt JC. Probabilistic Outputs for Support Vector Machines and Comparisons to Regularized Likelihood Methods. *Adv LARGE MARGIN Classif*. 1999:61--74. http://citeseerx.ist.psu.edu/viewdoc/summary?doi=10.1.1.41.1639. Accessed August 6, 2020.

4. Elkan C. The Foundations of Cost-Sensitive Learning. January 2001.

5. Zhao Y, Chen F, Zhai R, et al. Correction for population stratification in random forest analysis. *Int J Epidemiol*. 2012;41(6):1798-1806. doi:10.1093/ije/dys183

6. Chyzhyk D, Varoquaux G, Thirion B, Milham M. Controlling a confound in predictive models with a test set minimizing its effect. *2018 Int Work Pattern Recognit Neuroimaging, PRNI 2018*. July 2018. doi:10.1109/PRNI.2018.8423961

7. Steyerberg EW. *Clinical Prediction Models*. 2nd ed. Springer International Publishing; 2019.

8. Austin PC, Steyerberg EW. Graphical assessment of internal and external calibration of logistic regression models by using loess smoothers. *Stat Med*. 2014;33(3):517-535. doi:10.1002/sim.5941
