# Supplementary material for: Machine learning for prediction of schizophrenia using genetic and demographic factors in the UK biobank
Source: Schizophr Res. 2022 Aug;246:156–64. doi: 10.1016/j.schres.2022.06.006 (PMC9399753; doi:10.1016/j.schres.2022.06.006)
Supplement: Appendix B — Supplementary results. [file mmc2.docx]

## Appendix B: supplementary results

#### Sample
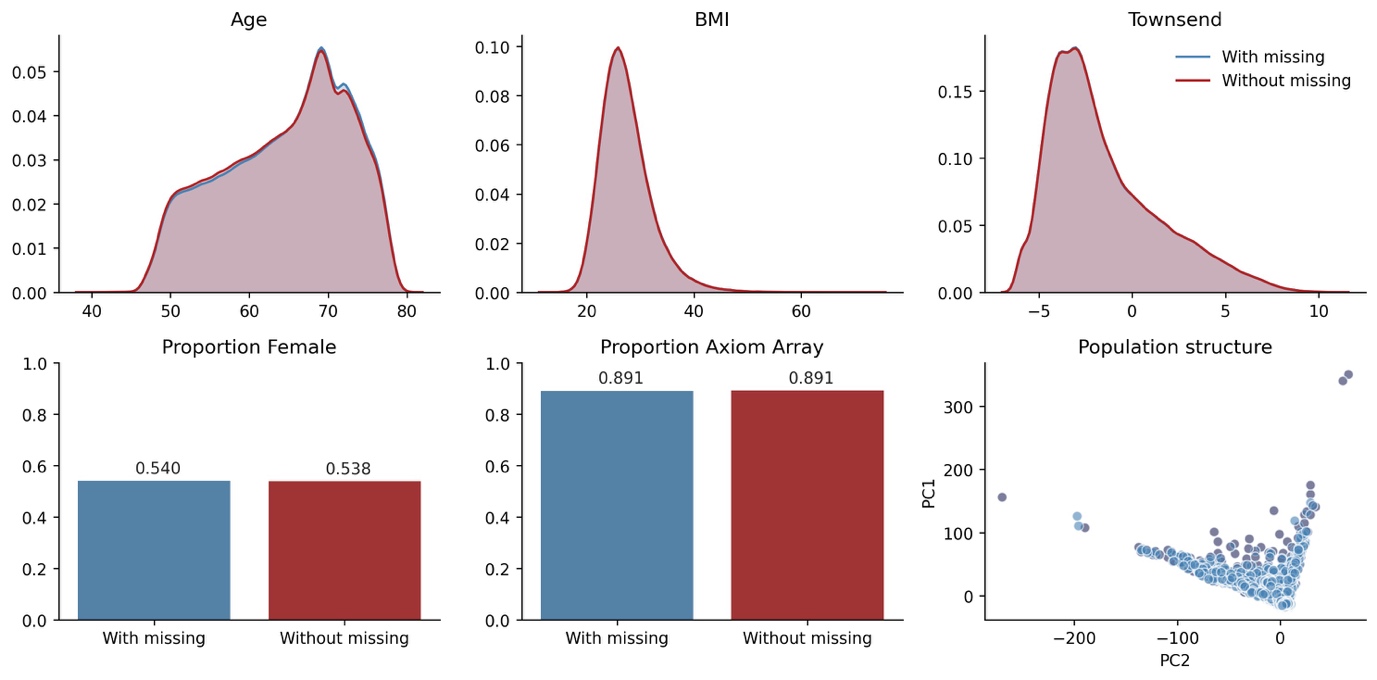


**Figure S2: Comparison of controls before and after exclusion by missingness**. Townsend show the Townsend deprivation index. Population structure is shown using the first 2 principal components provided by UK Biobank. Comparisons are shown for all controls after quality control but before subsampling. A sample size of 370,468 controls was reduced to 341,774 after exclusion by missingness.


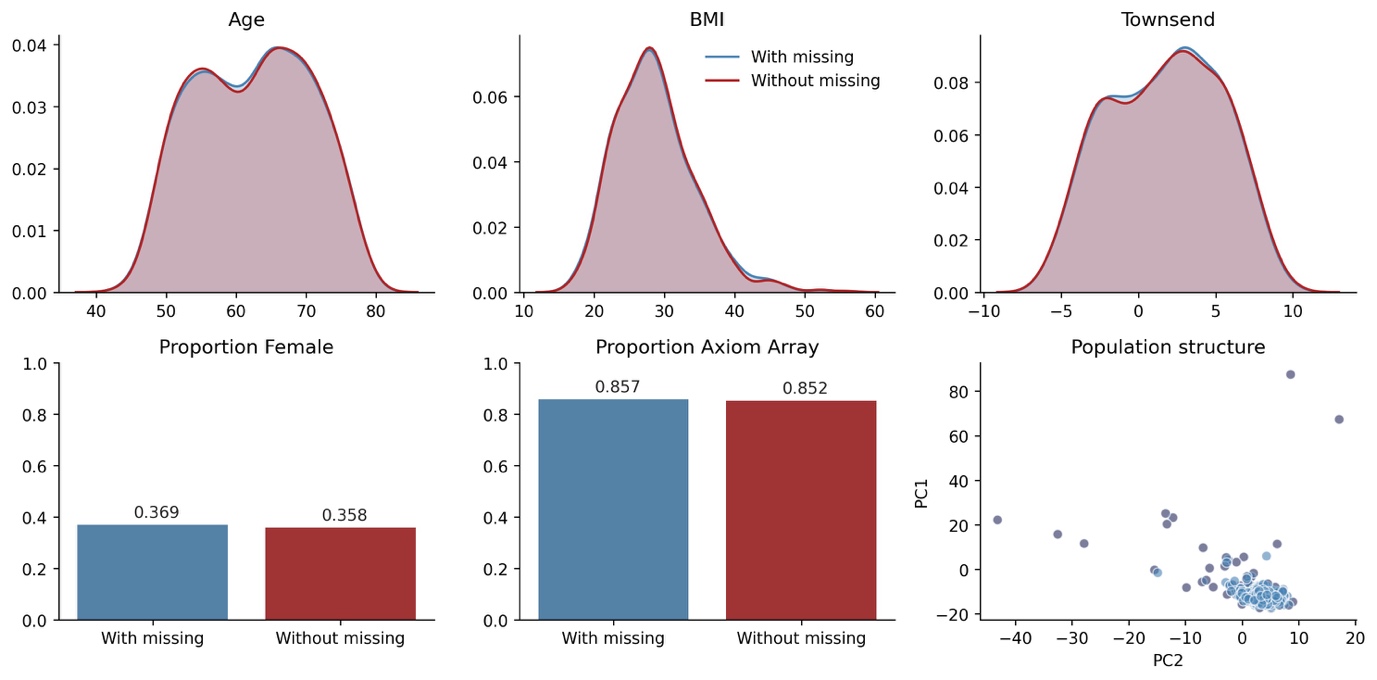


**Figure S3: Comparison of cases before and after exclusion by missingness.** 807 cases before exclusions were reduced to 738 after exclusion by missingness in demographic predictors.

####

**Figure S4: Per-predictor missingness split by cases and controls.** No missingness was present in winter birth or sex. Bars are annotated with sample size while the proportion is shown on the *x* axis. Number of siblings, parental depression and qualifications appear to show some difference in missingness between cases and controls.

| **Predictor** | **Test** | **Test-statistic** | **P-value (uncorrected)** |
| --- | --- | --- | --- |
| Winter birth | None |  |  |
| Sex | None |  |  |
| Handedness | Fisher’s exact | 0 | 1 |
| Number of siblings | Chi-squared | 17.33 | 0.000031 |
| Parental depression | Chi-squared | 8.80 | 0.0030 |
| Qualifications | Chi-squared | 2.04 | 0.15 |

**Table S2: Tests for differential missingness in demographic predictors.** Number of siblings and parental depression show nominally significant differential missingness between cases and controls, though proportion of records complete by predictor (Figure S4) and comparisons of basic characteristics in cases and controls by missingness (Figures S2 and S3) indicate the reduced (complete-case) sample is largely representative of the full cohort.

#### Importance scores


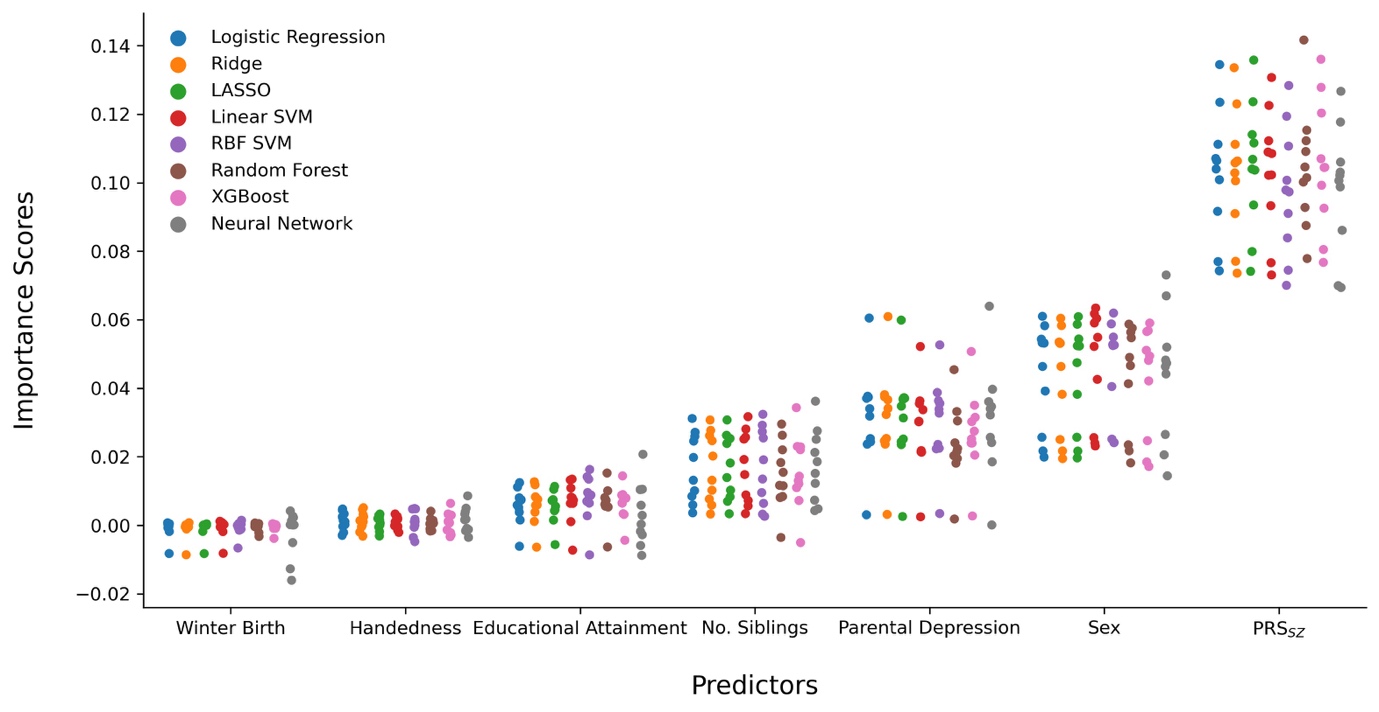


**Figure S5:** **Importance scores by classifiers**. Permutation feature importance for combined models showed similar importance scores.


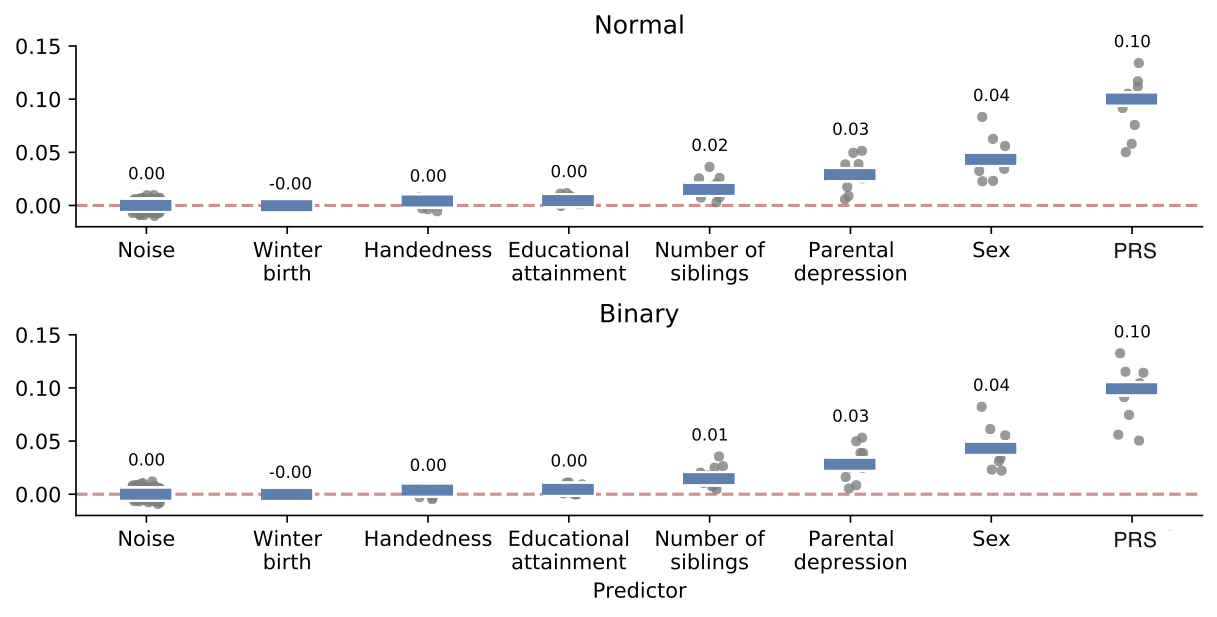


**Figure S6: Inclusion of noise predictors in importance scores for a logistic regression**. Nested cross-validation using a logistic regression was repeated with an associated “noise” predictor included as part of the model, where variables were drawn from Gaussian or Binomial distributions. Permutation feature importance gave an average score of 0 to unassociated predictors.

| **Variable** | **Beta** | ***p*-value** | **Lower 95% CI** | **Upper 95% CI** | **Direction** |
| --- | --- | --- | --- | --- | --- |
| Intercept | -2.343 | 1.5E-31 | -2.736 | -1.950 | n/a |
| Winter Birth | -0.004 | 9.7E-01 | -0.199 | 0.191 | + [1] |
| Handedness | -0.340 | 4.7E-03 | -0.576 | -0.104 | - [2] |
| Sex | 0.744 | 3.0E-17 | 0.572 | 0.917 | + [3] |
| Number of siblings | 0.372 | 4.7E-06 | 0.213 | 0.532 | + [4] |
| Parental depression | 0.841 | 7.9E-17 | 0.643 | 1.039 | + [5] |
| Educational Attainment | -0.252 | 4.8E-03 | -0.427 | -0.077 | - [6] |
| PRS | 0.546 | 1.4E-32 | 0.456 | 0.636 | + [7] |
| c01 | -0.008 | 4.0E-01 | -0.025 | 0.010 | n/a |
| c02 | 0.001 | 9.6E-01 | -0.024 | 0.025 | n/a |
| c03 | -0.006 | 7.4E-01 | -0.042 | 0.030 | n/a |
| c04 | -0.002 | 8.0E-01 | -0.021 | 0.016 | n/a |
| c05 | 0.004 | 4.7E-01 | -0.007 | 0.016 | n/a |
| c06 | 0.023 | 2.7E-01 | -0.018 | 0.065 | n/a |
| c07 | -0.007 | 6.4E-01 | -0.036 | 0.022 | n/a |
| c08 | 0.003 | 8.6E-01 | -0.031 | 0.037 | n/a |
| c09 | -0.012 | 2.3E-01 | -0.032 | 0.008 | n/a |
| c10 | 0.033 | 1.3E-01 | -0.010 | 0.076 | n/a |
| c11 | -0.024 | 1.6E-01 | -0.057 | 0.010 | n/a |
| c12 | -0.040 | 7.1E-02 | -0.084 | 0.003 | n/a |
| c13 | -0.047 | 7.9E-02 | -0.099 | 0.005 | n/a |
| c14 | -0.020 | 1.4E-01 | -0.046 | 0.006 | n/a |
| c15 | 0.019 | 4.0E-01 | -0.026 | 0.065 | n/a |
| array | 0.287 | 2.0E-02 | 0.045 | 0.528 | n/a |

**Table S3: Direction of effect for associations with schizophrenia.** A logistic regression was fit to the nested sample of 1:5 cases-to-controls including the first 15 principal components and genotyping array as covariates, as importance scores do not show direction of effect. PRS was standardised to mean 0 and unit variance before analysis; all other variables are coded as described in the main text. Direction column gives the known direction of effect in the literature, given the same coding used here (main text, section 2.2 – predictors). Only winter birth shows a different direction of effect, but is not significantly associated with schizophrenia in this dataset. Pseudo-*R^2^*: 0.1, log-likelihood ratio *p*-value: 4.68x10^-72^.

#### Resampling

####
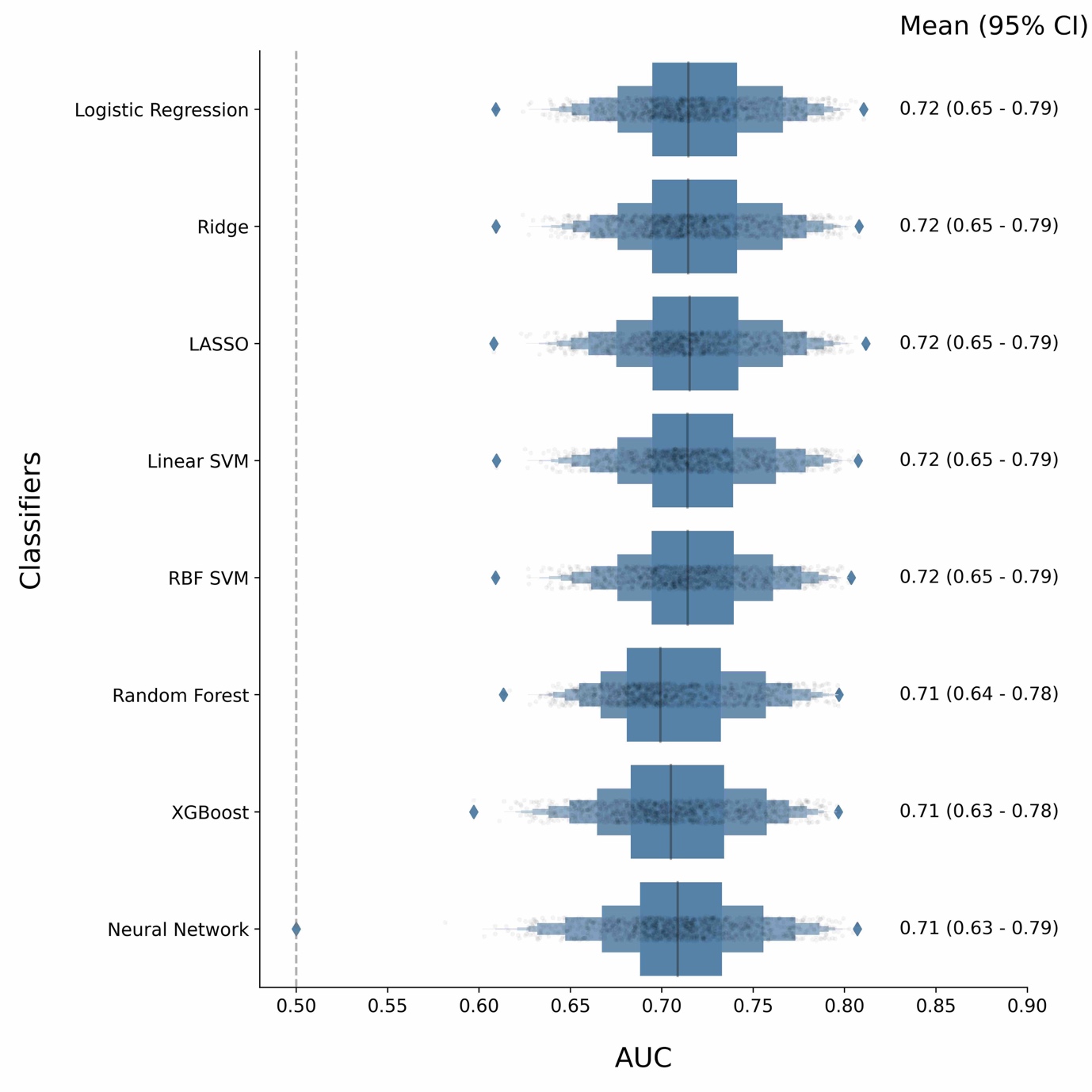


**Figure S7: resampling controls**. Models combining all predictors and using a case-control ratio of 1:5 were re-run 100 times, each time randomly re-drawing the 3,690 controls from the UK Biobank, with replacement. Results indicate reported values in Figure 2 are reflective of the average obtained from resampling and that distributions for each classifier overlap greatly.


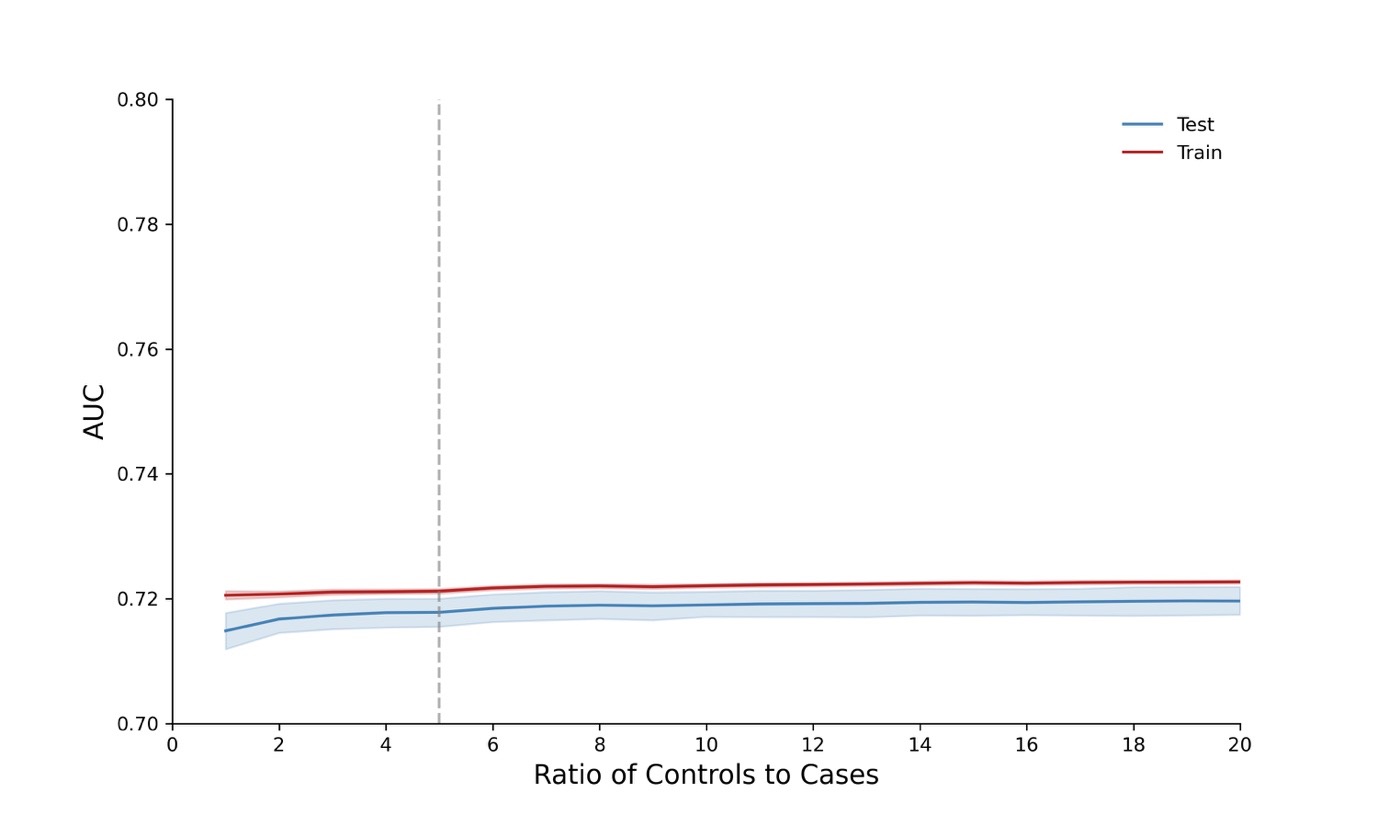


**Figure S8: Learning curve analysis.** Application of logistic regression to nested case-control designs of 1 to 20x controls show similar average train and test AUC for a ratio of 2:1 or above. For each ratio of cases-to-controls, controls were resampled from the full UK Biobank cohort 100 times with bootstrap 95% confidence intervals shown over the 100 repeats. Learning curve analysis was restricted to logistic regression to reduce computation time, but is likely similar across classifiers as results for logistic regression are highly consistent across case-control ratios and resampling of controls. Together with previous recommendations[8], the learning curve analysis indicates a ratio of 1:5 cases-to-controls is a reasonable trade-off between the increase in computational burden and AUC with the sampling fraction.

#### Precision-Recall

|  | PRS*_SZ_* | | | Clinical/Demographic | | | All | | |
| --- | --- | --- | --- | --- | --- | --- | --- | --- | --- |
| Classifier | Mean | Lower 95 | Upper 95 | Mean | Lower 95 | Upper 95 | Mean | Lower 95 | Upper 95 |
| LR | 0.282 | 0.261 | 0.303 | 0.307 | 0.277 | 0.337 | 0.353 | 0.319 | 0.386 |
| Ridge | 0.282 | 0.261 | 0.303 | 0.307 | 0.277 | 0.337 | 0.353 | 0.319 | 0.386 |
| LASSO | 0.282 | 0.261 | 0.303 | 0.306 | 0.276 | 0.335 | 0.353 | 0.319 | 0.386 |
| Linear SVM | 0.282 | 0.261 | 0.303 | 0.302 | 0.267 | 0.337 | 0.352 | 0.319 | 0.384 |
| RBF SVM | 0.287 | 0.263 | 0.311 | 0.306 | 0.275 | 0.337 | 0.352 | 0.320 | 0.384 |
| RF | 0.281 | 0.260 | 0.302 | 0.295 | 0.268 | 0.323 | 0.333 | 0.300 | 0.365 |
| XGBoost | 0.264 | 0.248 | 0.281 | 0.293 | 0.263 | 0.322 | 0.332 | 0.300 | 0.363 |
| NN | 0.281 | 0.260 | 0.302 | 0.304 | 0.274 | 0.333 | 0.348 | 0.313 | 0.382 |

**Table S4: AUPRC for all classifiers**. Mean is the average across 10 outer folds of nested cross-validation. Results were computed using “average_precision_score” in scikit-learn for each fold and classifier.

#### Calibration

Assessment of whether predicted probabilities from the nested sample could be successfully applied to the whole of UK Biobank showed that all classifiers overestimate schizophrenia risk (Figure S8b), but that correction for the control sampling fraction of 0.01 significantly improved calibration (Figure S8c). Assessment for strong calibration[9] indicates that models are well-calibrated within each level of categorical predictors (Figures S9-S13).


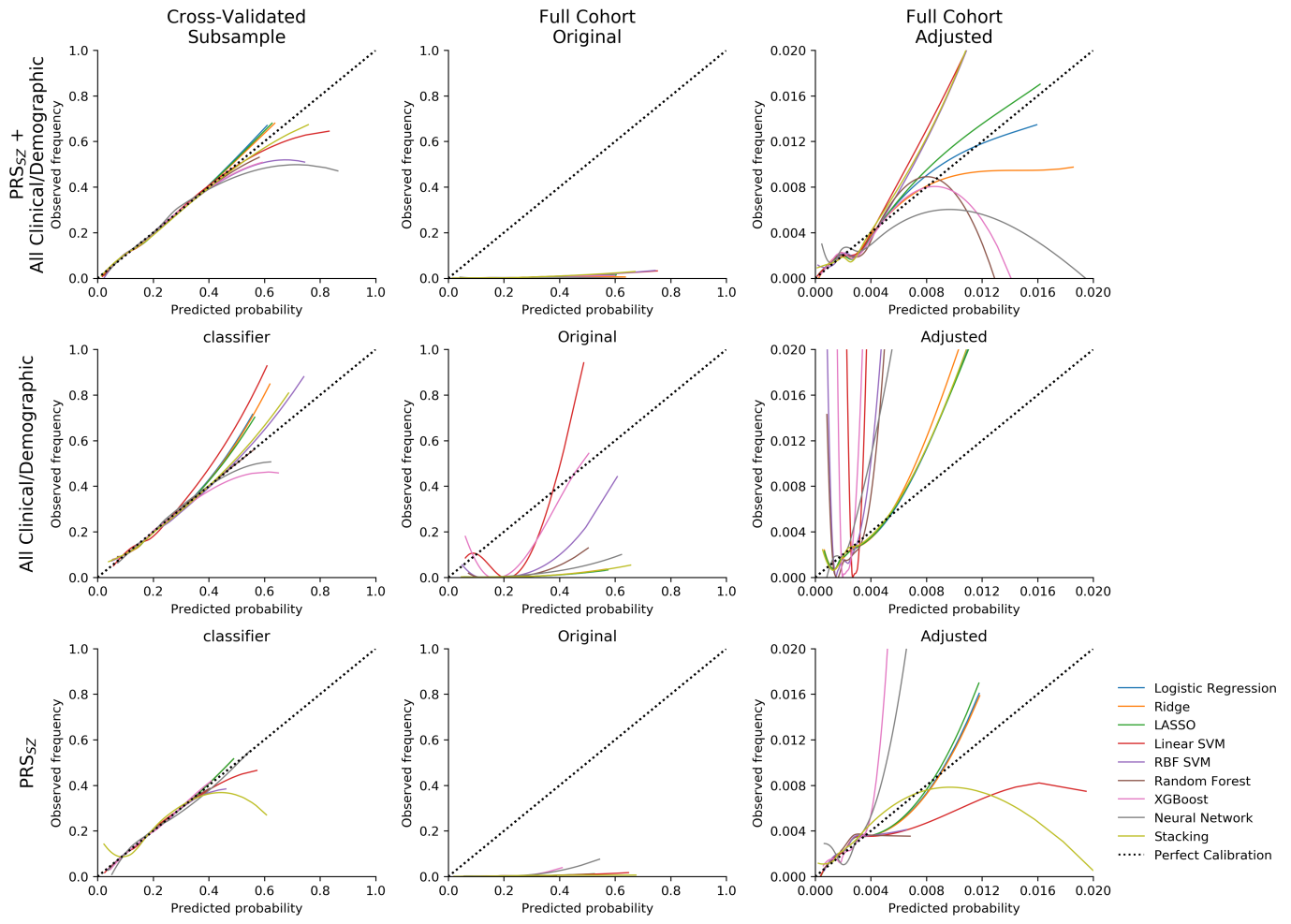


**Figure S9: Overall calibration with adjustment for the sampling fraction.** Calibration was assessed by looking at combined models in the nested case-control sample (a). Investigation of calibration in all observations (b) was conducted by combining refit predictions in remaining controls with those in the nested design. Adjusting predicted probabilities for the sampling fraction improves calibration, which is expected to be better where discrimination is higher and to vary more as curves move toward the upper right[10]. Loess smoothers were estimated on a maximum subsample of 30,000 participants due to the large memory requirements of the “loess” function in the scikit-misc package being greater than those available on the HPC cluster.


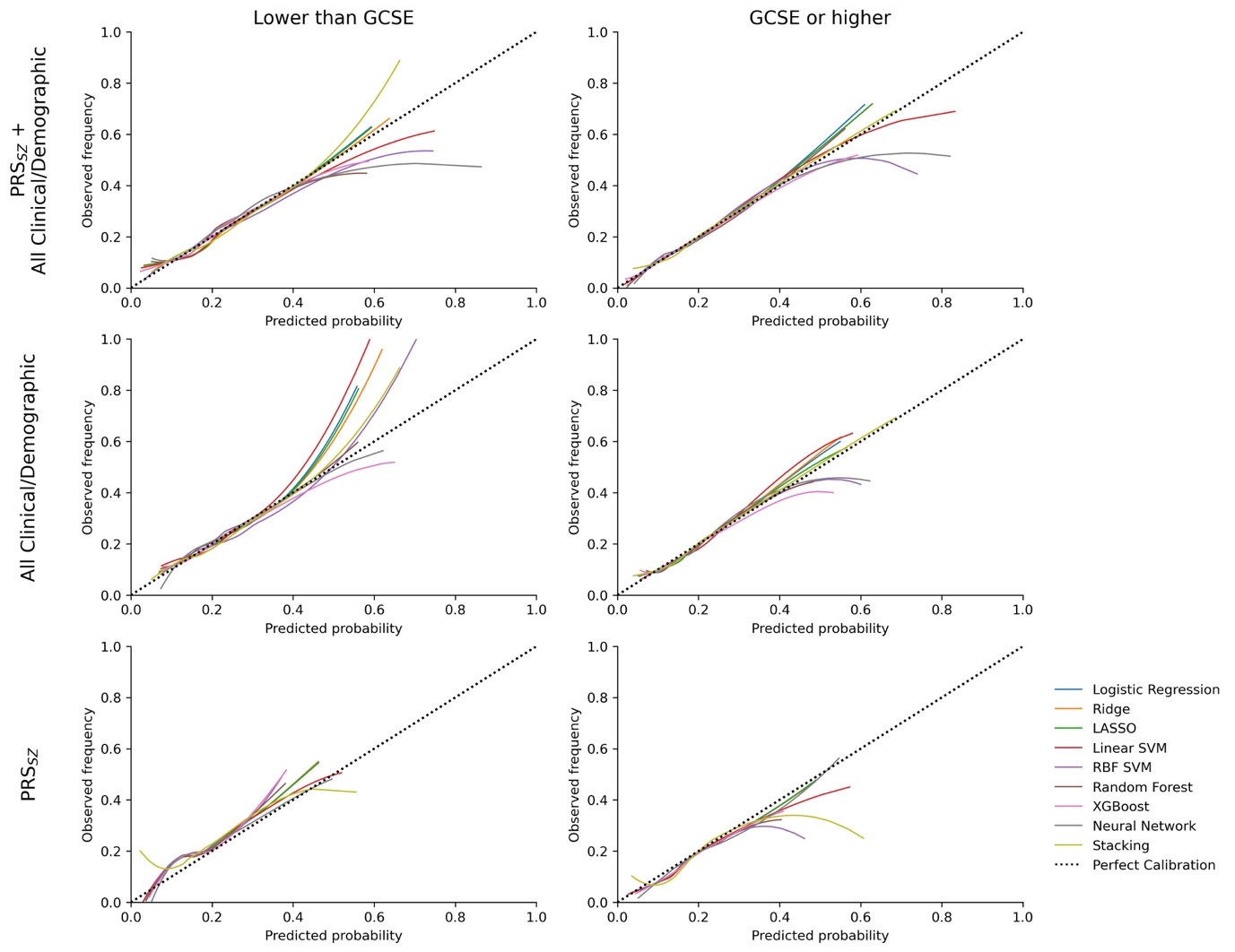


**Figure S10: Calibration by educational attainment.** Calibration split by levels of educational attainment (coded as 1 for GCSE and higher, and 0 for below) shows good calibration across all levels and classifiers in the nested case-control design.


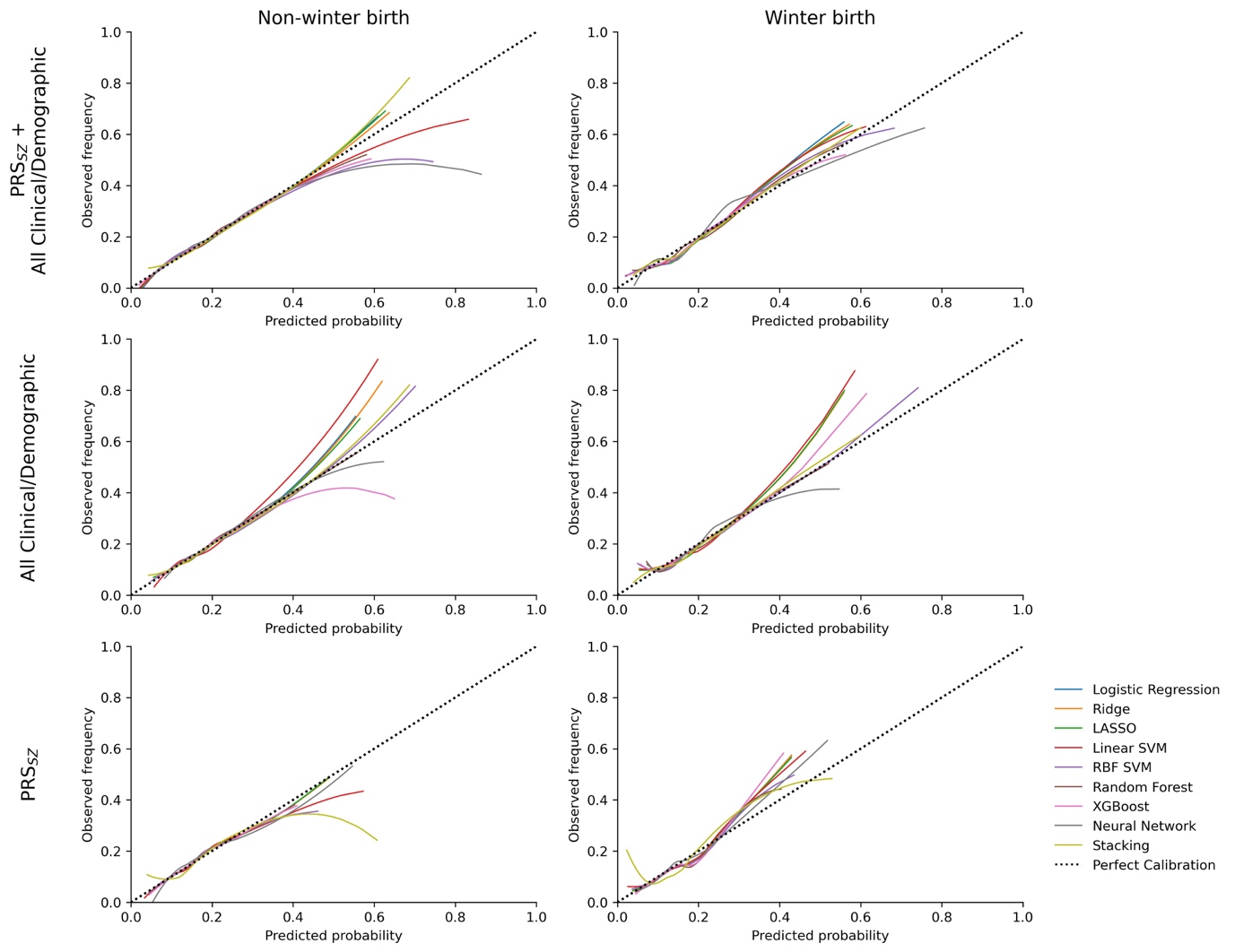


**Figure S11: Calibration by winter birth.** Calibration split by levels of winter birth shows good calibration across all levels and classifiers in the nested case-control design.

**
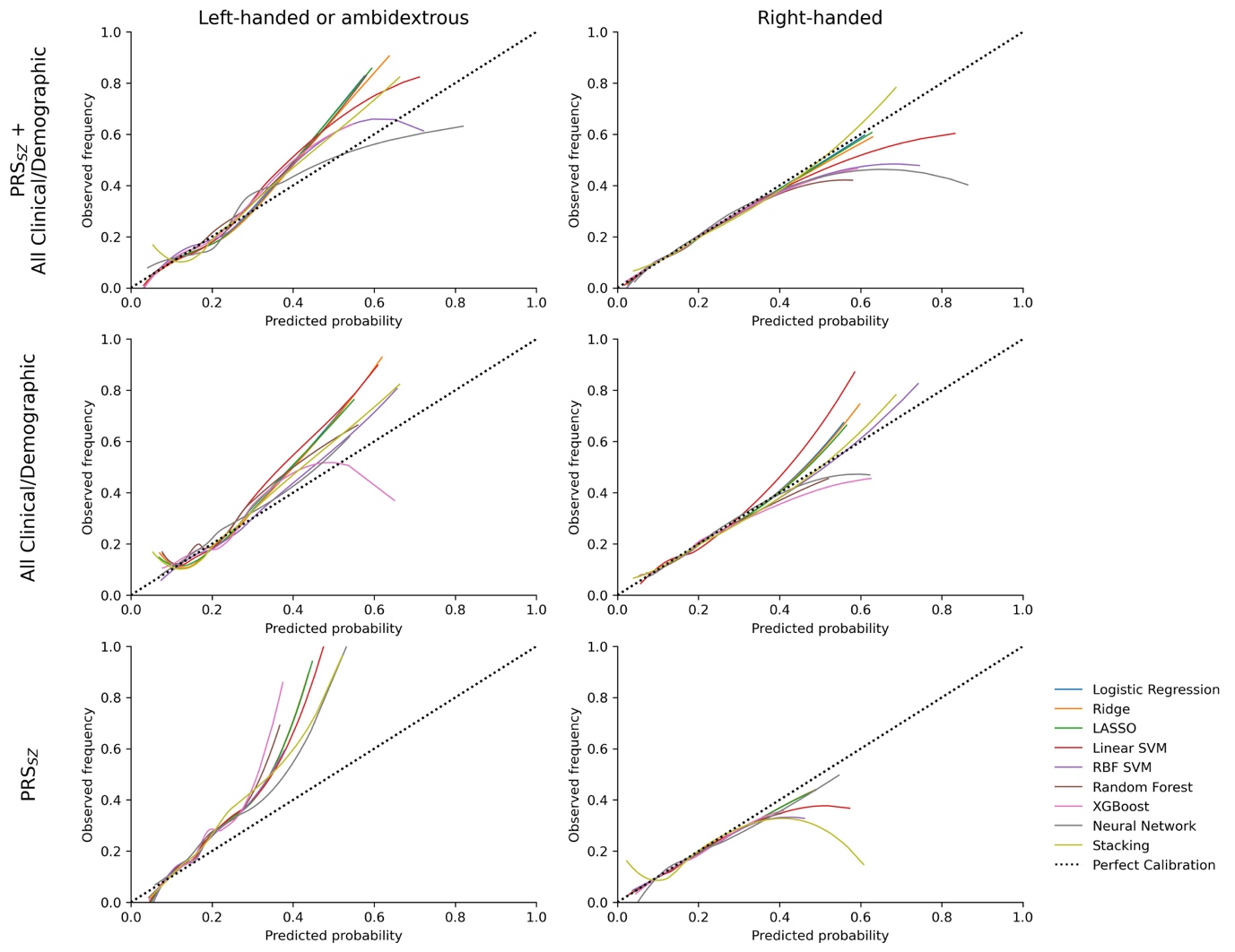
**

**Figure S12: Calibration by handedness.** Calibration split by levels of handedness in all models; small deviations from diagonal can be observed for left-handed or ambidextrous, particularly for PRS-only where there is a slight underestimation of risk in left/ambidextrous participants, but most curves follow the diagonal; worse calibration is expected where number of observations is lower such as in left-handed or ambidextrous (*n* = 548) compared to right-handed (*n* = 3,880).

**
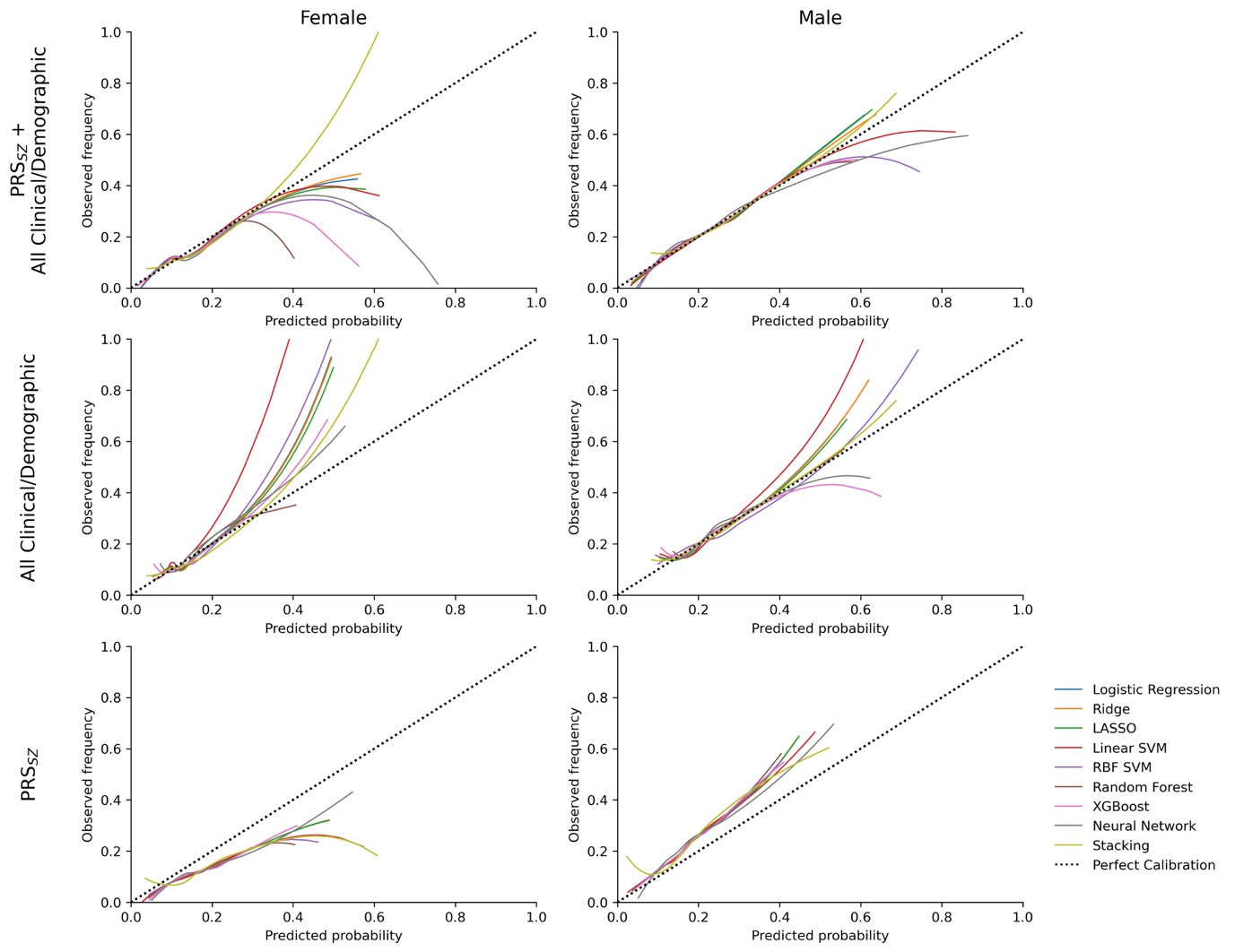
**

**Figure S13: Calibration by sex.** Calibration is good with deviations expected as curves move toward the upper right. PRS shows slight underestimation of risk in males and overestimation of risk in females, but inclusion of sex as a predictor in the original model (top two rows) shows better calibration.


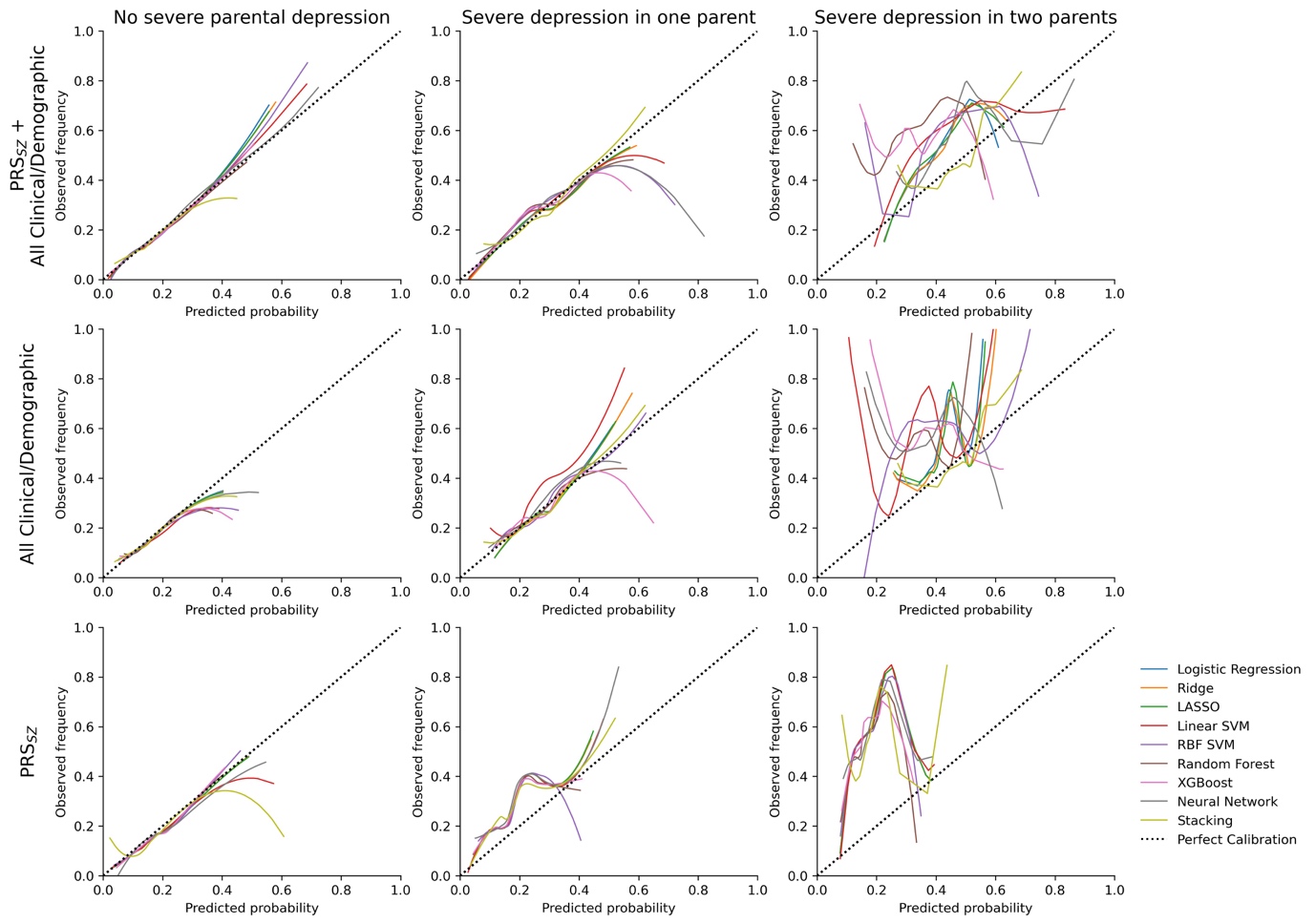


**Figure S14: Calibration by parental depression**. Calibration is good for categories with a larger number of events, but is difficult to assess for depression in both parents as confidence intervals are extremely large (not shown) and number of events is low (*n* = 36). PRS shows worse calibration for depression in a single parent.

#### Assessment of model predictions

ML*_SZ_*, evaluated through a cross-validated beta regression in remaining UK Biobank controls (Figure 3), showed the highest mean *R^2^* for deprivation (maximum *R^2^*=0.014, Linear SVM, all clinical/demographic model), smoking status (*R^2^*=0.018, XGBoost, all clinical/demographic), BMI (*R^2^*=0.016, XGBoost, all clinical/demographic) and cognitive testing, in particular fluid intelligence (*R^2^*=0.026, Linear SVM, all clinical/demographic) and digit symbol substitution (*R^2^*=0.01, RBF SVM, PRS*_SZ_* and all clinical/demographic combined). In controls, average effect sizes across beta regression models showed higher BMI and deprivation, and being a smoker, to be associated with higher predicted risk of schizophrenia by LR and ML*_SZ_* models, while better performance on fluid intelligence and digit symbol substitution was associated with lower predicted risk (Table S4).

| Primary Predictors | Secondary Predictors | LR | Ridge | LASSO | Linear SVM | RBF SVM | RF | XGB | NN | Stacking |
| --- | --- | --- | --- | --- | --- | --- | --- | --- | --- | --- |
| PRS*_SZ_*+ All Clinical/Demographic | BMI | 0.039 | 0.041 | 0.037 | 0.042 | 0.042 | 0.038 | 0.039 | 0.064 | 0.039 |
|  | Digit Symbol Substitution | -0.055 | -0.057 | -0.053 | -0.055 | -0.055 | -0.053 | -0.057 | -0.051 | -0.051 |
|  | Deprivation | 0.050 | 0.052 | 0.047 | 0.050 | 0.051 | 0.043 | 0.048 | 0.055 | 0.048 |
|  | Fluid Intelligence | -0.062 | -0.065 | -0.058 | -0.063 | -0.066 | -0.052 | -0.060 | -0.040 | -0.058 |
|  | Smoking Status | 0.066 | 0.069 | 0.064 | 0.067 | 0.066 | 0.060 | 0.063 | 0.075 | 0.065 |
| All Clinical/Demographic | BMI | 0.056 | 0.055 | 0.055 | 0.008 | 0.038 | 0.045 | 0.012 | 0.047 | 0.051 |
|  | Digit Symbol Substitution | -0.043 | -0.043 | -0.043 | -0.006 | -0.030 | -0.035 | -0.009 | -0.038 | -0.040 |
|  | Deprivation | 0.053 | 0.052 | 0.052 | 0.007 | 0.037 | 0.033 | 0.011 | 0.043 | 0.057 |
|  | Fluid Intelligence | -0.047 | -0.048 | -0.045 | -0.008 | -0.038 | -0.026 | -0.010 | -0.030 | -0.053 |
|  | Smoking Status | 0.065 | 0.064 | 0.064 | 0.008 | 0.042 | 0.049 | 0.013 | 0.055 | 0.063 |
| PRS*_SZ_* | BMI | -0.012 | -0.012 | -0.012 | -0.012 | -0.012 | -0.011 | -0.010 | -0.009 | -0.010 |
|  | Digit Symbol Substitution | -0.020 | -0.020 | -0.020 | -0.019 | -0.020 | -0.019 | -0.017 | -0.015 | -0.018 |
|  | Deprivation | 0.005 | 0.005 | 0.005 | 0.005 | 0.005 | 0.005 | 0.004 | 0.004 | 0.005 |
|  | Fluid Intelligence | -0.024 | -0.024 | -0.024 | -0.024 | -0.024 | -0.023 | -0.020 | -0.018 | -0.021 |
|  | Smoking Status | 0.012 | 0.012 | 0.012 | 0.012 | 0.012 | 0.011 | 0.010 | 0.009 | 0.010 |

**Table S5: mean estimated effect sizes from a cross-validated beta regression.** All variables were standardised within cross-validation as models aimed to maximise prediction. Models were trained to predict scores which had been assigned to each individual by machine learning models trained on the original (primary) predictors. Models therefore took the form Risk*_SZ-ML_* ~ secondary predictor, where Risk*_SZ-ML_* is the calibrated risk scores given by ML models. Effect sizes are shown for secondary predictors with the highest mean test-fold *R^2^*.


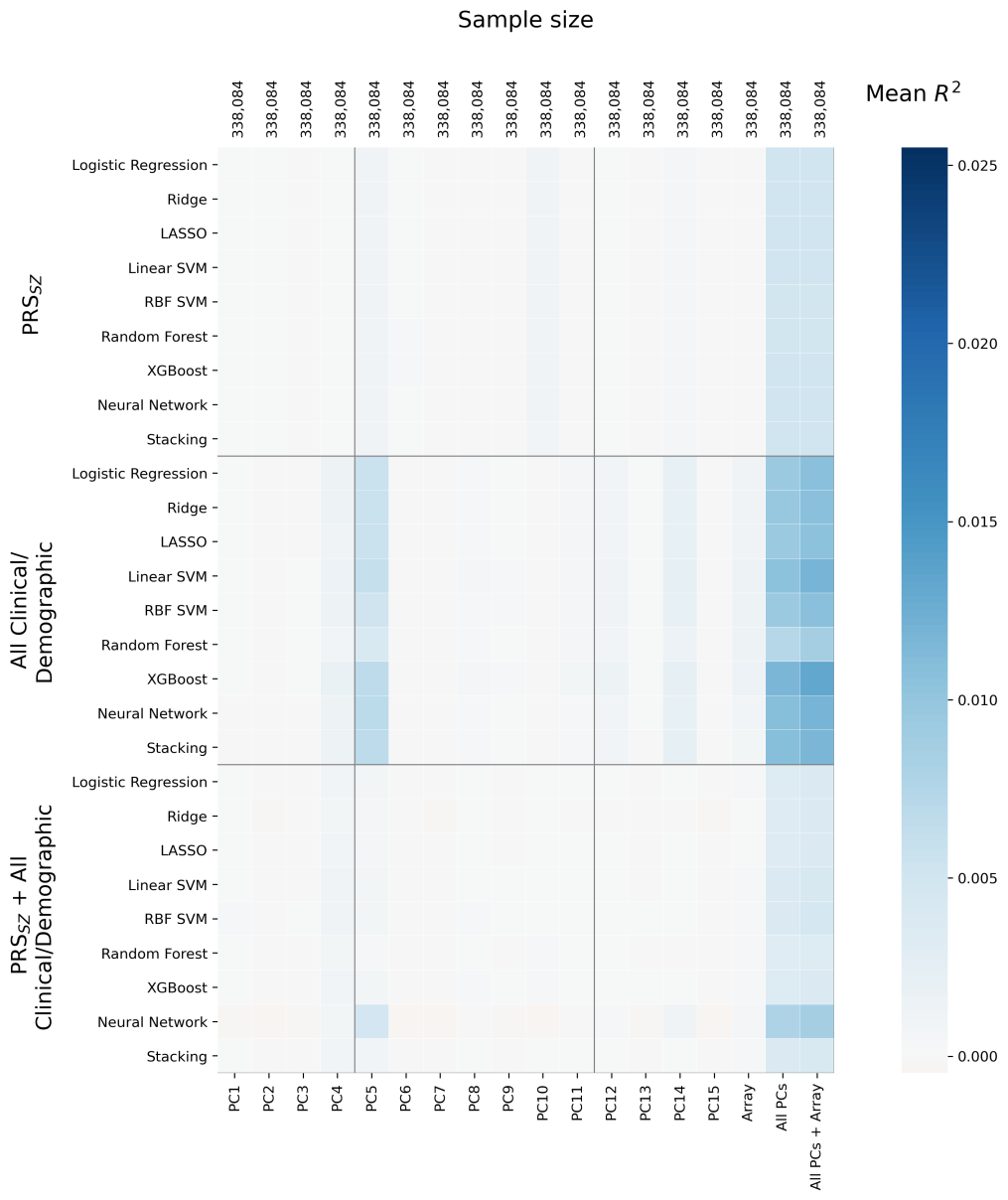


**Figure S14: Assessment of deconfounding procedures in remaining controls.** Analysis used a cross-validated beta regression, as described in Figure 3, such that higher *R^2^* indicates variables on the x-axis are better at explain predictions from models listed on the y-axis. Results show some residual confounding remaining in predictions from PRS*_SZ_* only. This is expected given that deconfounding procedures are run within train/test folds and not on the whole sample before splitting, as the former is more appropriate in a prediction modelling context, meaning the remaining 338,084 controls have a broader distribution when visualised by principal components (PCs) which is not captured when regressing-off PCs in the training data. Higher variance is explained in predictions from models using clinical/demographic predictors only, which were not adjusted for PCs in model development.

**References**

1. Qeoffrey Davies by, Welham J, Chant D, Fuller Torrey E, McQrath J. A Systematic Review and Meta-analysis of Northern Hemisphere Season of Birth Studies in Schizophrenia Downloaded from.

2. Dragovic M, Hammond G. Handedness in schizophrenia: a quantitative review of evidence. Acta Psychiatr Scand 2005; 111: 410–419.

3. McGrath J, Saha S, Chant D, Welham J. Schizophrenia: A Concise Overview of Incidence, Prevalence, and Mortality. Epidemiol Rev 2008; 30: 67–76.

4. Wahlbeck K, Osmond C, Forsén T, Barker DJP, Eriksson JG. Associations between childhood living circumstances and schizophrenia:</br>a population-based cohort study. Acta Psychiatr Scand 2001; 104: 356–360.

5. Radua J, Ramella-Cravaro V, Ioannidis JPA, Reichenberg A, Phiphopthatsanee N, Amir T, et al. What causes psychosis? An umbrella review of risk and protective factors. World Psychiatry 2018; 17: 49–66.

6. MacCabe JH, Lambe MP, Cnattingius S, Torrång A, Björk C, Sham PC, et al. Scholastic achievement at age 16 and risk of schizophrenia and other psychoses: a national cohort study. Psychol Med 2008; 38: 1133–1140.

7. Purcell SM, Wray NR, Stone JL, Visscher PM, O’Donovan MC, Sullivan PF, et al. Common polygenic variation contributes to risk of schizophrenia and bipolar disorder. Nature 2009; 460: 748–752.

8. Biesheuvel CJ, Vergouwe Y, Oudega R, Hoes AW, Grobbee DE, Moons KGM. Advantages of the nested case-control design in diagnostic research. BMC Med Res Methodol 2008; 8: 1–7.

9. Steyerberg EW. Clinical Prediction Models2nd ed.Springer International Publishing; 2019.

10. Austin PC, Steyerberg EW. Graphical assessment of internal and external calibration of logistic regression models by using loess smoothers. Stat Med 2014; 33: 517–535.
